# Supplementary material for: Genome-wide association study of Alzheimer’s disease CSF biomarkers in the EMIF-AD Multimodal Biomarker Discovery dataset
Source: Transl Psychiatry. 2020 Nov 22;10:403. doi: 10.1038/s41398-020-01074-z (PMC7680793; doi:10.1038/s41398-020-01074-z)

**Supplementary Methods**

**Alzheimer's Disease Neuroimaging Initiative (ADNI) sample**

ADNI started in 2003 as a public-private collaboration under the supervision of Principle Investigator Michael W. Weiner, MD. The primary goal of ADNI is to study whether serial magnetic resonance imaging (MRI), positron emission tomography (PET), other biological markers, and clinical and neuropsychological measures can be combined to measure the progression of mild cognitive impairment (MCI) and early Alzheimer’s disease (AD). Please see www.adni-info.org for the latest information. The ADNI participants utilized for our analyses originate from both ADNI1 and ADNIGo/2 and relate to those with available whole-genome sequencing (WGS) data. Accordingly, we label the subset of ADNI participants utilized here as “ADNI-WGS”.

**Genotype data handling, QC and imputation procedures**

Raw data processing, i.e. clustering and genotype calling from raw intensity data (idat format) was performed in GenomeStudio software (Illumina, Inc.) using the genotyping module (version 2.0.2). Samples with call rate <0.95 and p50GC <0.7 were excluded at this stage. We then used PLINK software (**v1.9;** [1]**)** to perform additional QC filtering, i.e. sex checks (--check-sex 0.25 0.75), strand check (--flip), missing genotype rate (--geno 0.02; --mind 0.05), Hardy-Weinberg equilibrium (HWE) tests (--hwe 0.000005), and minor allele frequency (MAF) filtering (--maf 0.01). For determining pairwise allele sharing (to identify cryptic relatedness), we used an LD-pruned set of markers (--indep-pairwise 1500 150 0.2). Pairwise allele-sharing IBD/IBS was determined using (--Z-genome --min 0.1). Overall, this procedure led to 498,589 QC-filtered SNPs in 931 samples suitable for imputation. The LD pruned dataset was also used for principal component analysis (PCA; using PLINK command ‘--pca') along with the reference dataset of the 1000 Genome Project Consortium Phase 3 (The 1000 Genomes Project Consortium, 2015) to assign ethnic descent groups using the five 1000G super-populations by k-nearest neighbor (k-NN; k=9) classification (using R package ‘class’ in R 2.3.2; [2]). This resulted in assigning a “European descent” to 898 out of all 931 samples; only these n=898 samples were used in the subsequent statistical analyses.

Before imputation, the QC’ed genotype data were then subjected to bcftools (v1.9) [3] for removing ambiguous SNPs, and flipping and swapping alleles to align to GRCh37/hg19. This was followed by haplotype phasing using SHAPEIT2 [4] and imputation of unobserved genotypes using Minimac3 [5] using a precompiled Haplotype Reference Consortium (HRC) reference panel (EGAD00001002729 including 39,131,578 SNPs from ~11K individuals). Post-imputation we only retained autosomal SNPs with minimac3 Rsq ≥0.3, MAF ≥1%, and HWE P-values ≥ 5E-6 (using best-guess genotypes in control subjects), leaving a total of 7,778,465 SNPs for statistical analyses.

**Polygenic risk score (PRS) analysis**

To aggregate data on multiple variants per individual we computed polygenic risk scores (PRS) for each individual which were then used as independent variable in the statistical analyses. Allele status and effect-size estimates were taken from the summary statistics of the largest AD GWAS published to date [6] and compared to the 2013 GWAS results from IGAP [7]. First, we removed ambiguous SNPs (A/T and C/G) from the list of considered variants. Furthermore, we only used SNPs with MAF >0.01 and imputation quality r2 >0.8 in the EMIF-AD MBD dataset. Next, LD pruning was performed on the CEU portion of the HRC reference panel used for imputation. To this end, we used PLINK 1.9 software [1] for two consecutive rounds of marker pruning (1^st^ round: --clump-p1 1 --clump-p2 1 --clump-r2 .5 --clump-kb 250; 2^nd^ round: --clump-p1 1 --clump-p2 1 --clump-r2 .2 --clump-kb 5000). Actual PRS were computed using the --score command in PLINK for a variety of P-value thresholds in the primary GWAS data (0.00000005, 0.000005, 0.0001, 0.01, 0.05, 0.10, 0.20, 0.30, 0.40, 0.50, 1.00). The resulting PRS were used as independent variable in the linear or logistic regression models adjusting for sex, age, and PC1 to PC5 as covariates. For the phenotypes not representing the diagnostic outcome, we also included diagnosis as additional covariate. For linear models, variance explained (r^2^) was derived from comparing results from the full model (including outcome phenotype and covariates) vs the null model (linear model with covariates only). For logistic models, we calculated Nagelkerke’s r^2^ using the R package fmsb.

**Supplementary References**

[1] S. Purcell *et al.*, “PLINK: a tool set for whole-genome association and population-based linkage analyses.,” *Am. J. Hum. Genet.*, vol. 81, no. 3, pp. 559–75, Sep. 2007.

[2] W. N. Venables and B. D. R. Springer, “Modern Applied Statistics with S Fourth edition.”

[3] V. Narasimhan *et al.*, “BCFtools/RoH: A hidden Markov model approach for detecting autozygosity from next-generation sequencing data,” *Bioinformatics*, 2016.

[4] O. Delaneau, J. Marchini, and J. F. Zagury, “A linear complexity phasing method for thousands of genomes,” *Nat. Methods*, 2012.

[5] S. Das *et al.*, “Next-generation genotype imputation service and methods,” *Nat. Genet.*, 2016.

[6] I. E. Jansen *et al.*, “Genome-wide meta-analysis identifies new loci and functional pathways influencing Alzheimer’s disease risk,” *Nat. Genet.*, p. 1, Jan. 2019.

[7] J.-C. Lambert *et al.*, “Meta-analysis of 74,046 individuals identifies 11 new susceptibility loci for Alzheimer’s disease,” *Nat. Genet.*, vol. 45, no. 12, pp. 1452–1458, Dec. 2013.

**Supplementary Tables**

Supplementary Tables 1-20 can be found in the MS-Excel file

“Supplementary_Tables.xls”.

**Supplementary Figures**

**All supplementary figures are arranged in the same format (“X” stands for figure number).**

**See Table 1 in the main text for a full description of variable names.**

Supplementary Figure XA: Manhattan (top) and quantile-to-quantile (bottom) plots of **SNP-based** genome-wide association results. Red line represents the threshold for genome-wide significance (α = 5.0E-08).

Supplementary Figure XB: Manhattan (top) and quantile-to-quantile (bottom) plots of **gene-based** genome-wide association results. Red line represents the threshold for genome-wide significance (α = 2.671E-06).

Gene-based plots drawn in FUMA.

**Supplementary Figure 1A.** AD vs NC


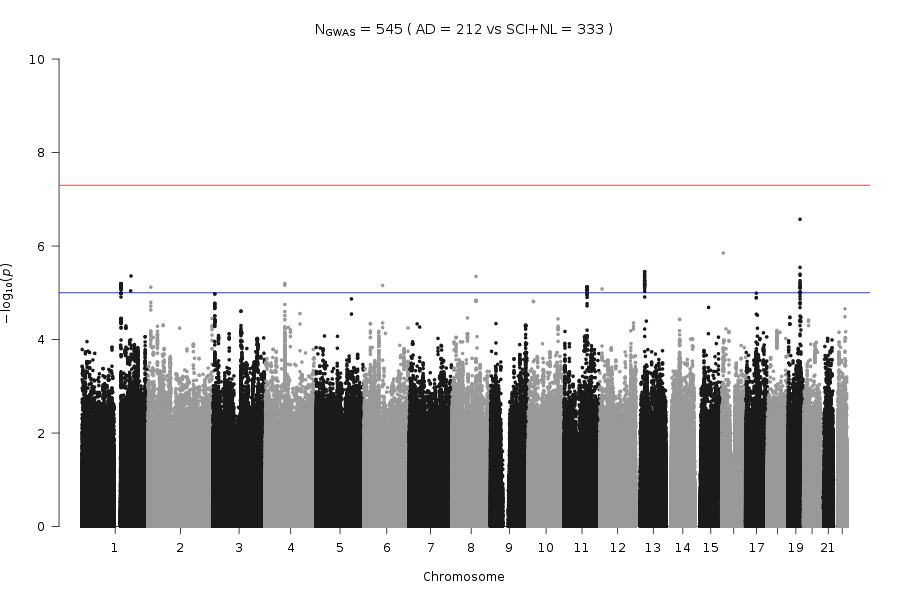


N_GWAS_ = 545 (AD = 212 vs NC = 333)


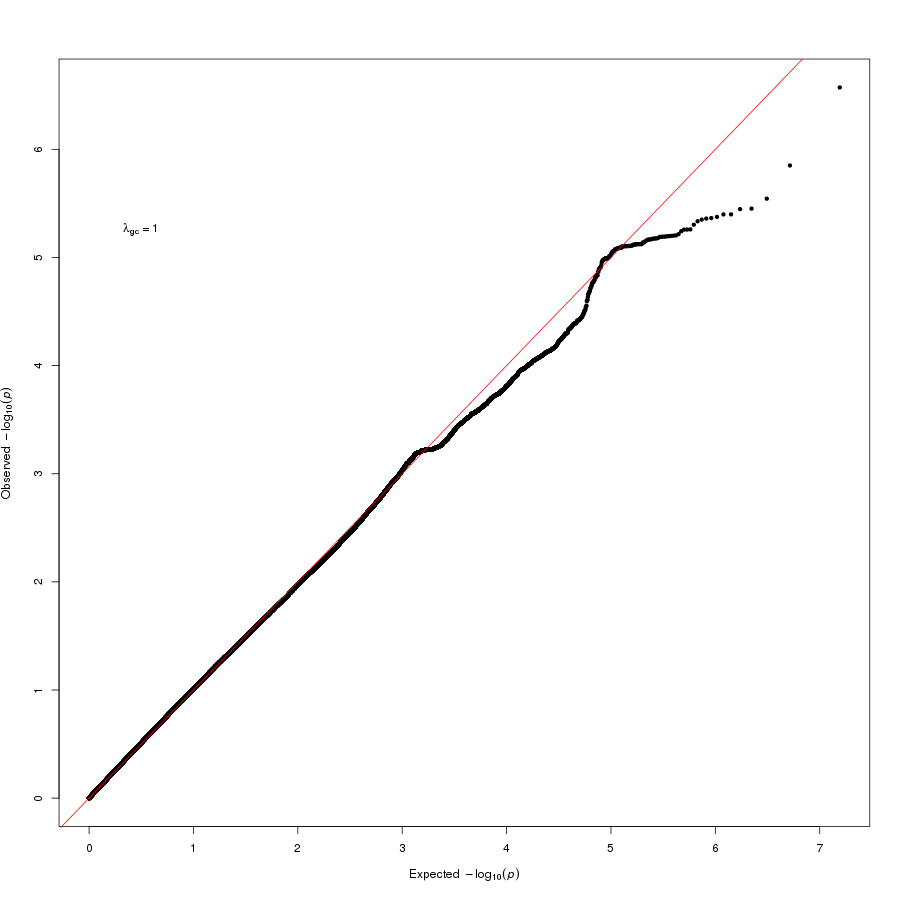


**Supplementary Figure 1B.** AD vs NC


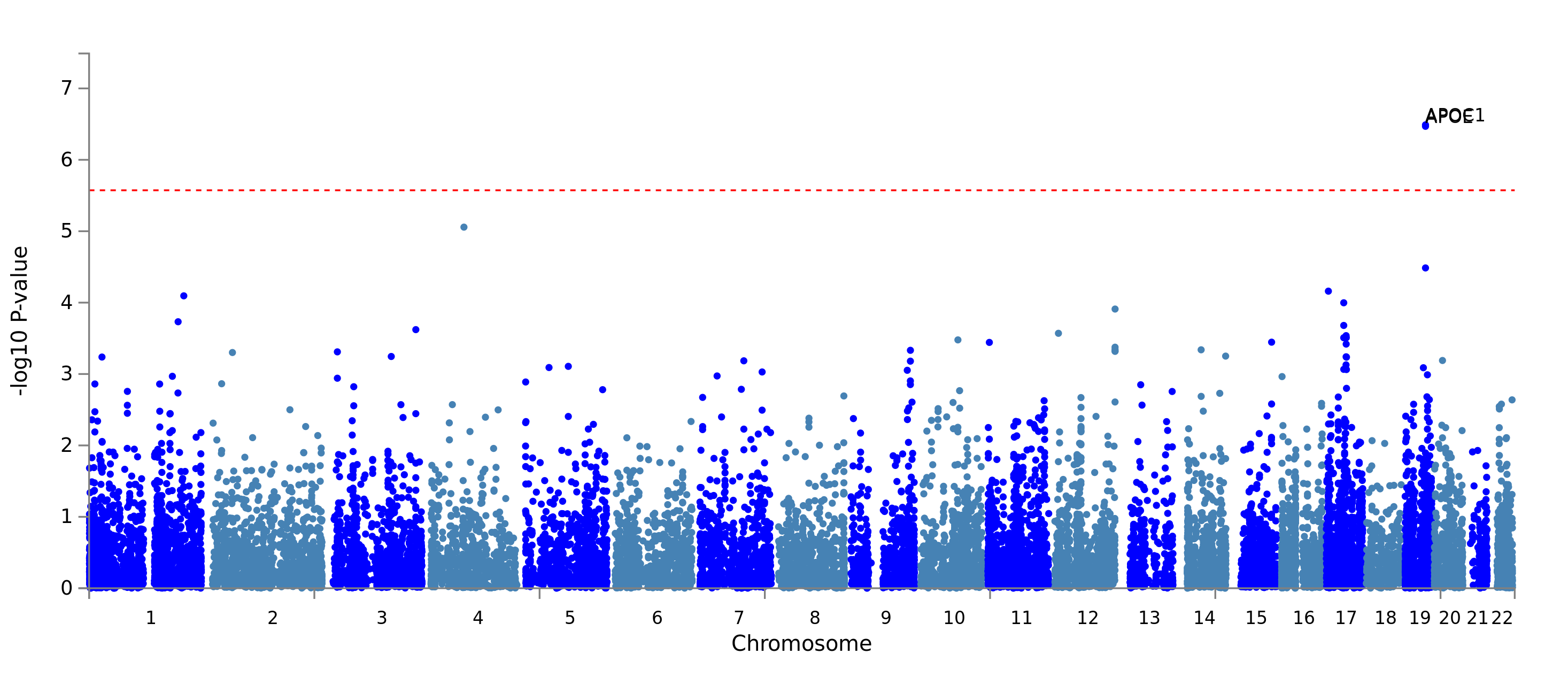


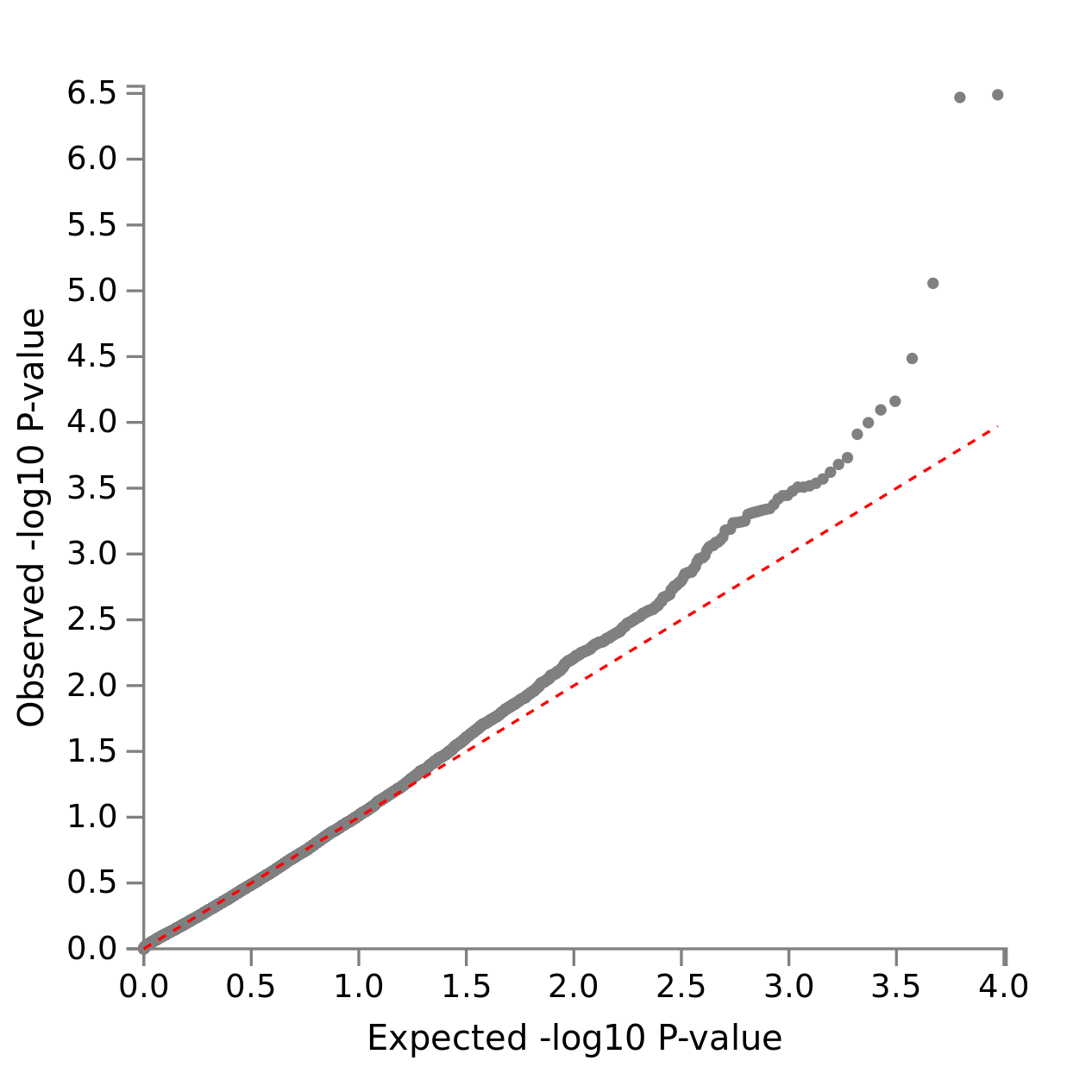


**Supplementary Figure 2A.**  MCI vs NC


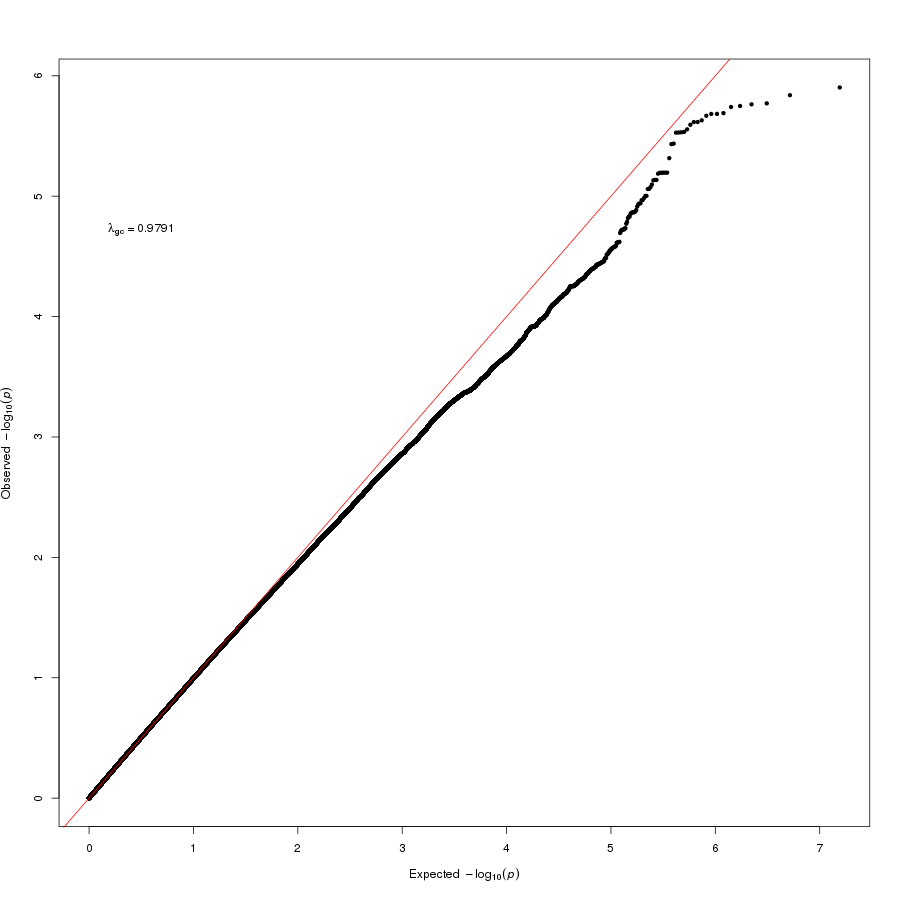

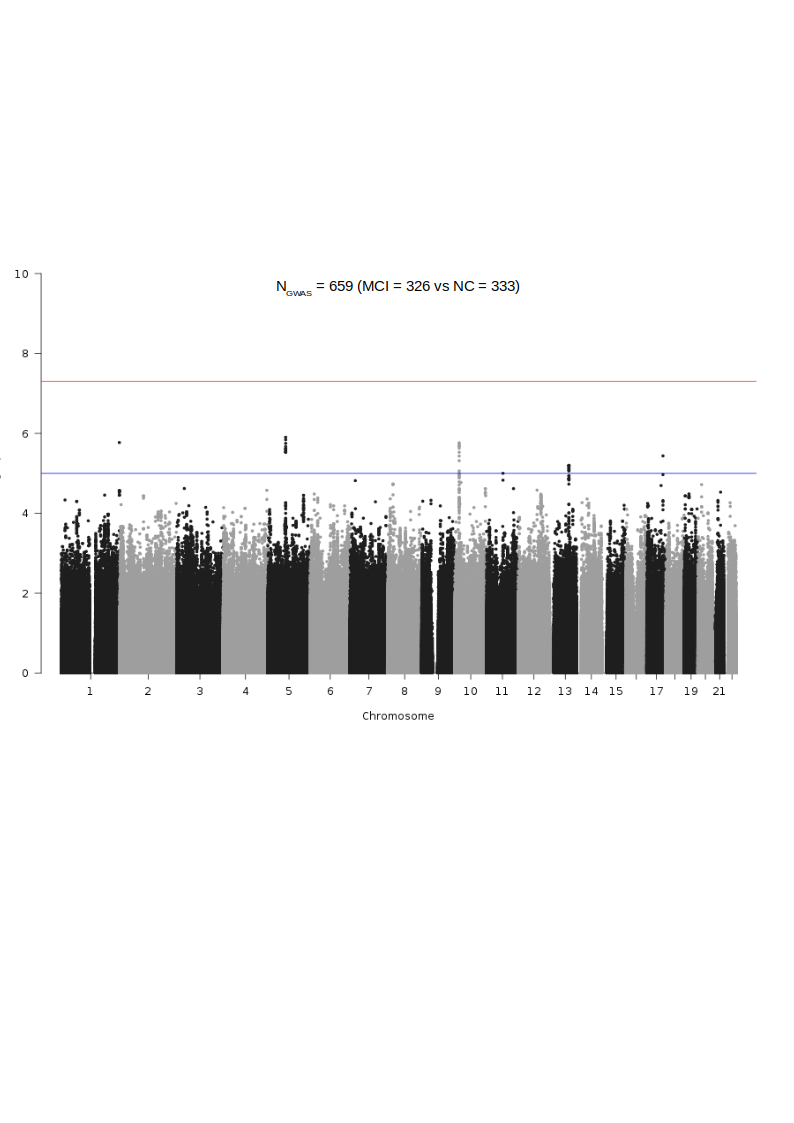


**Supplementary Figure 2B.** MCI vs NC


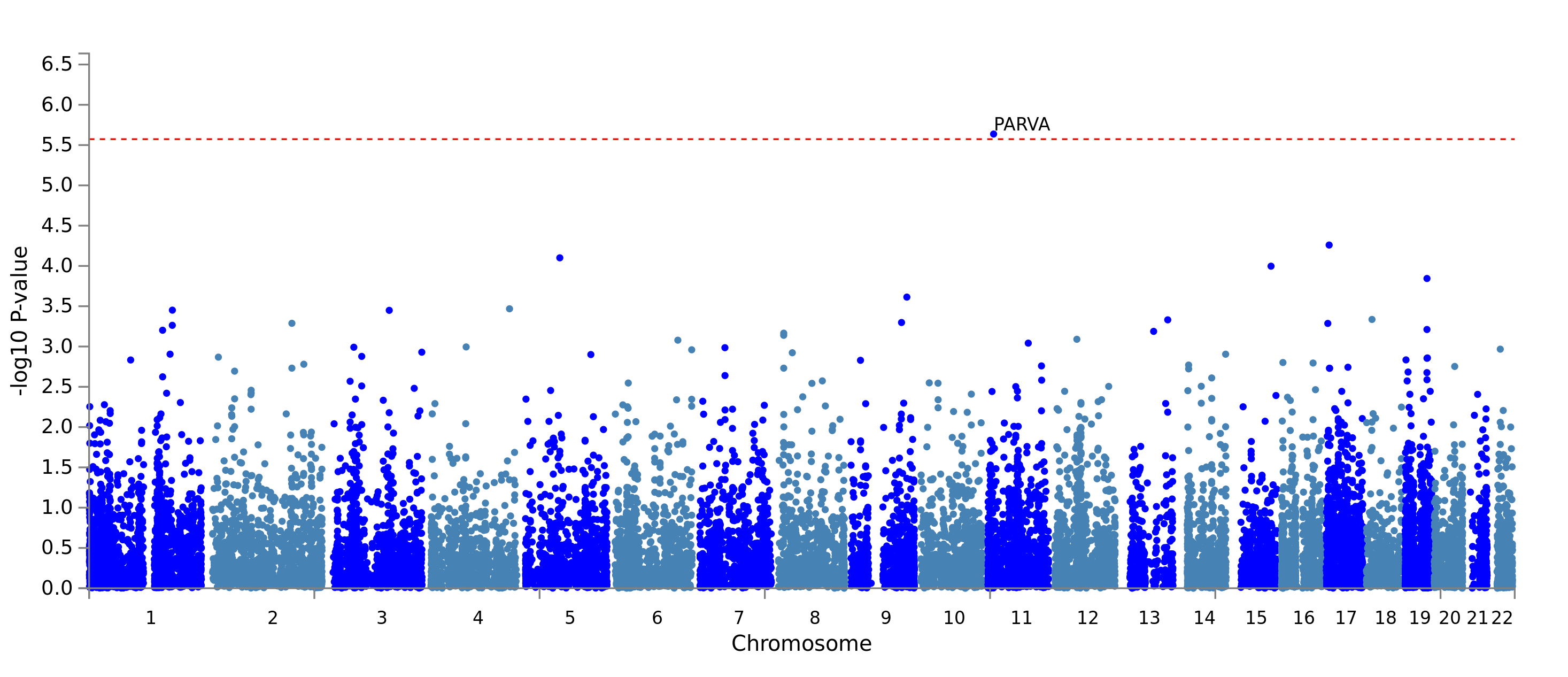


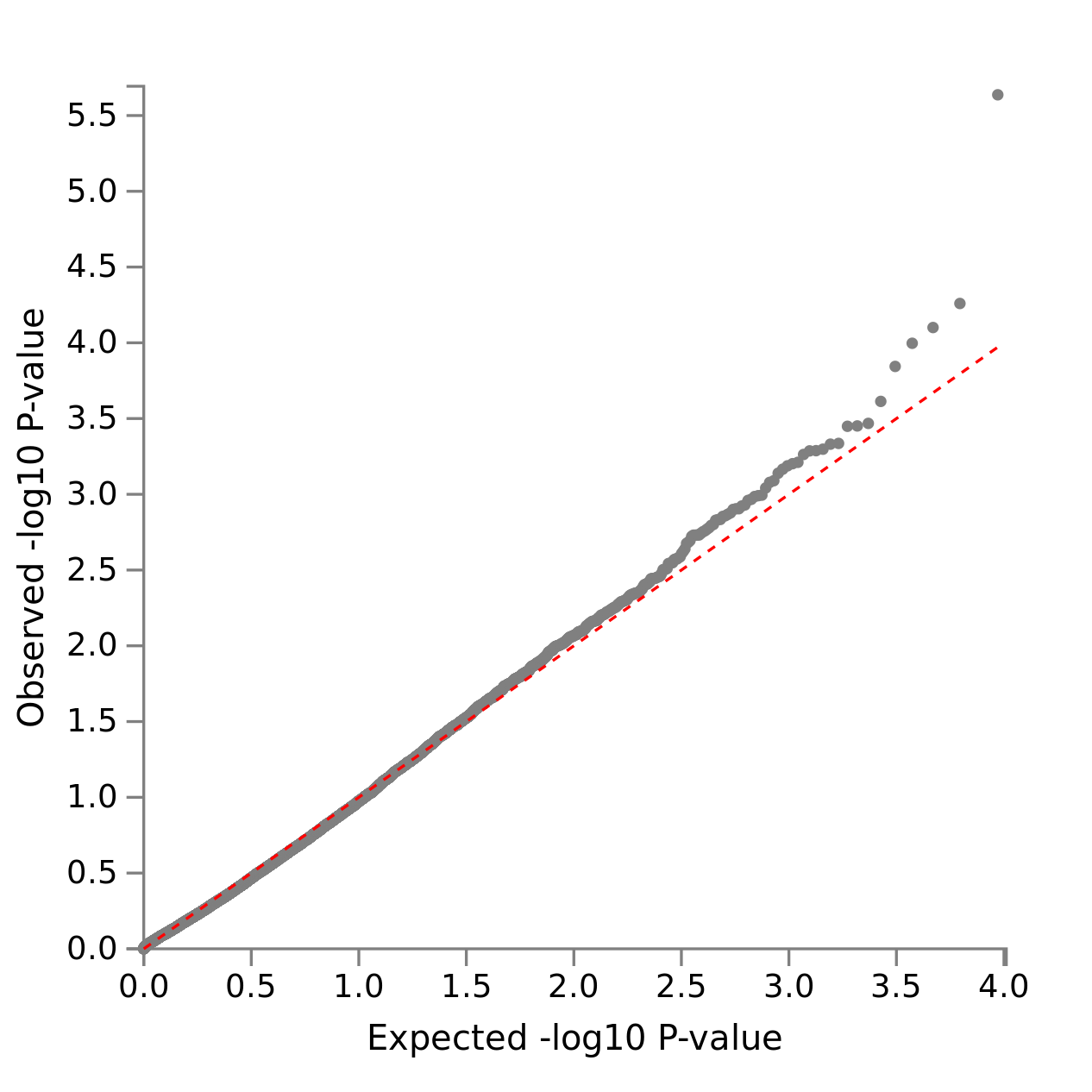


**Supplementary Figure 3A.** Central_CSF_ratiodich


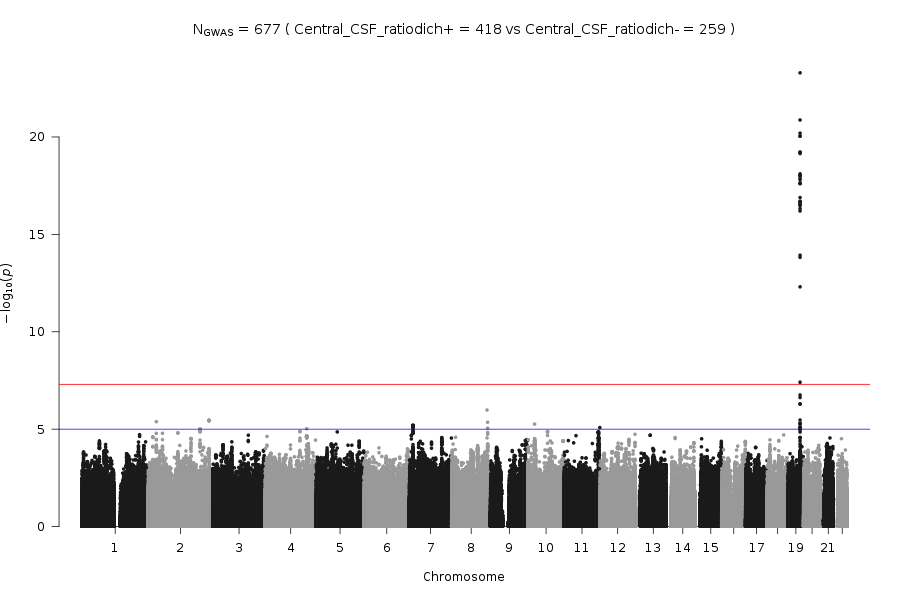


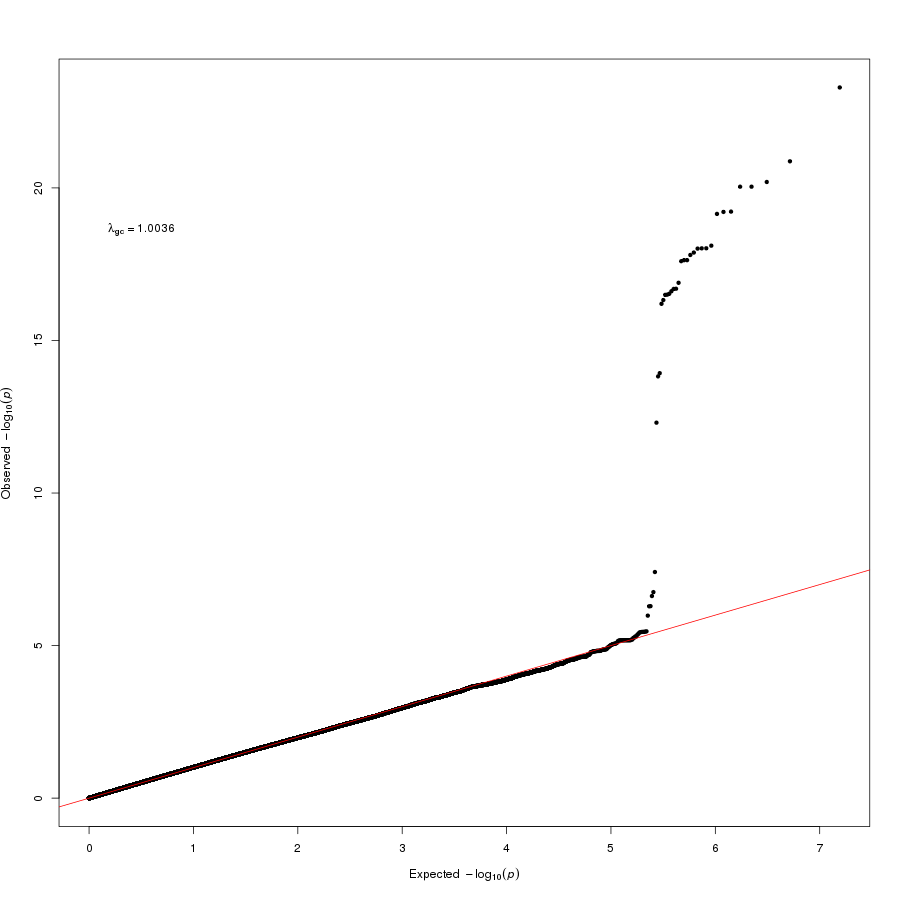


**Supplementary Figure 3B.** Central_CSF_ratiodich


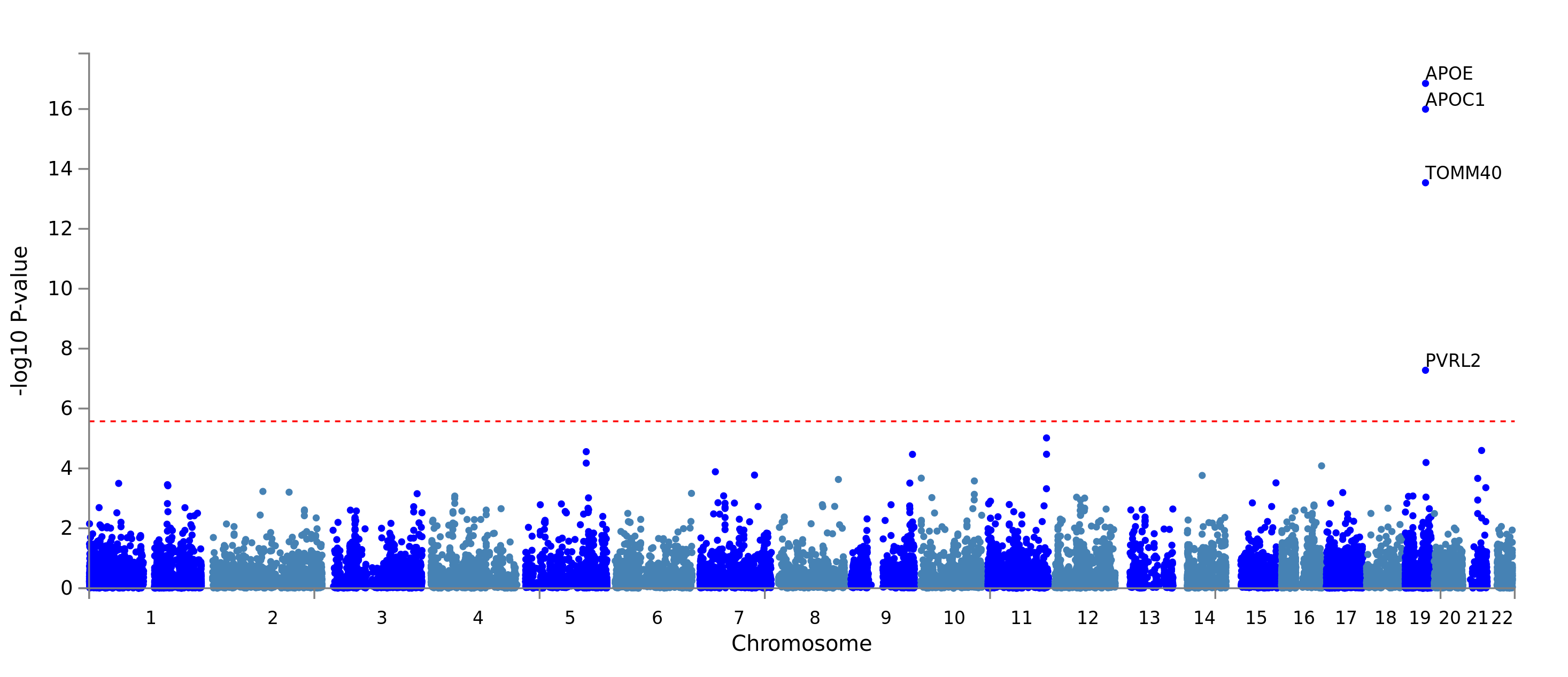


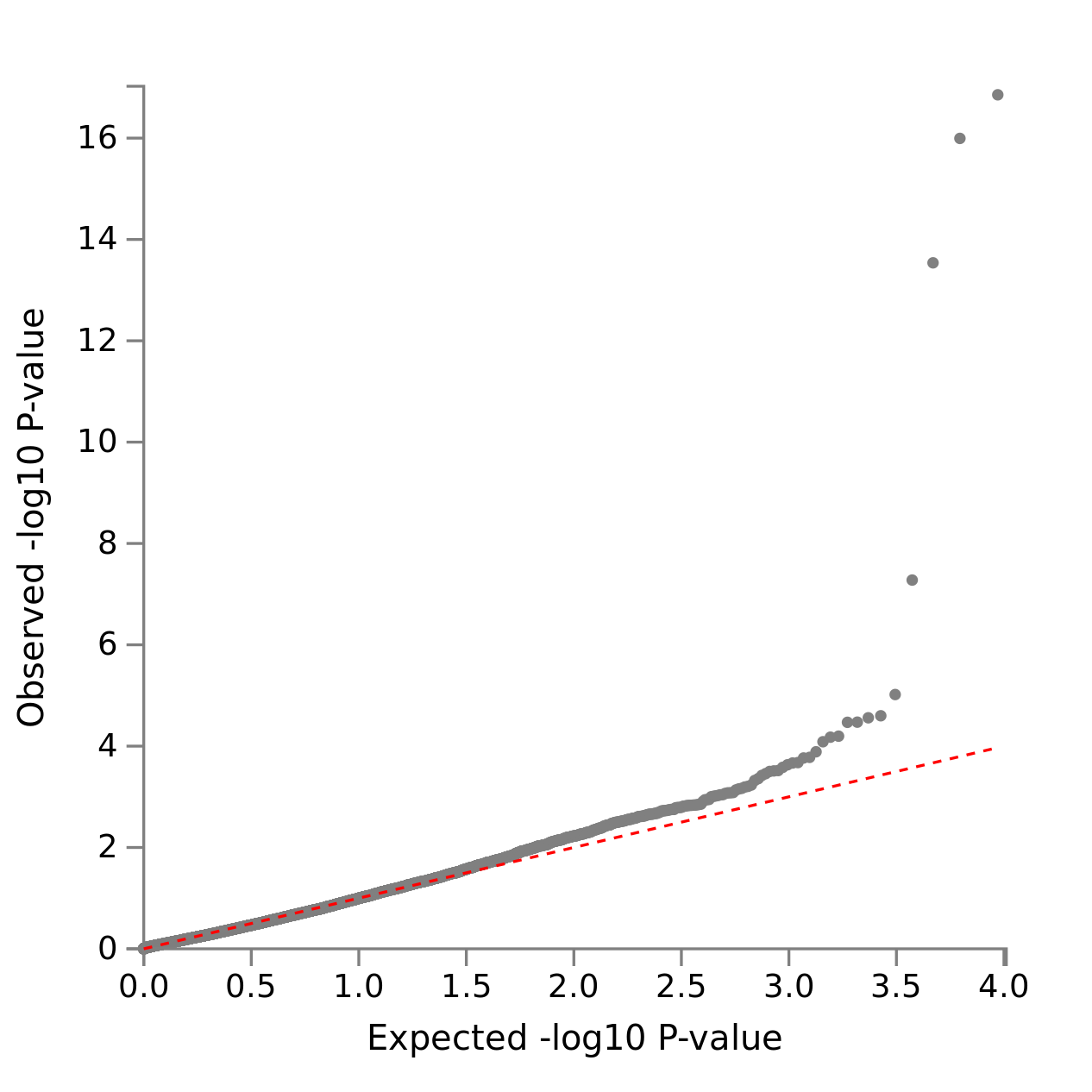


**Supplementary Figure 4A.** Local_AB42_Abnormal


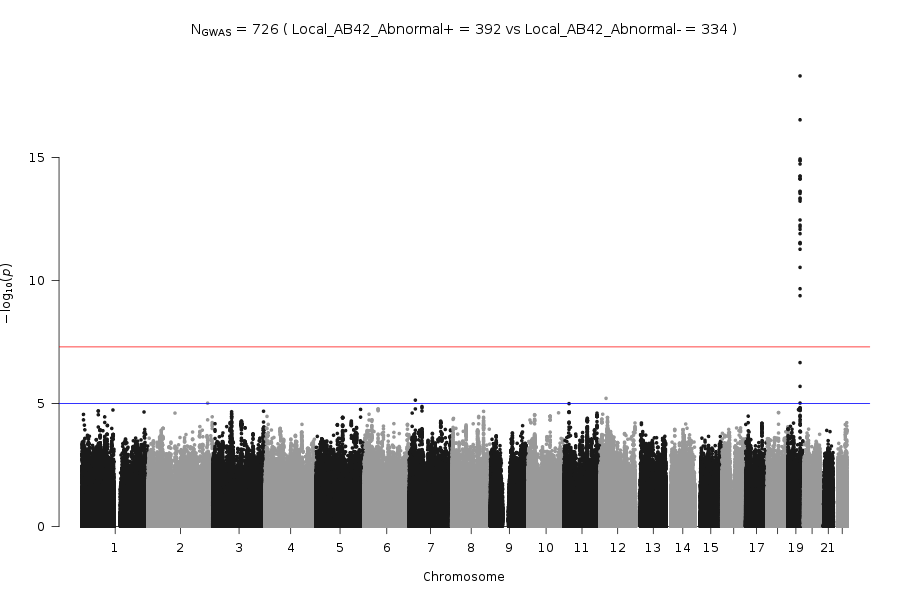


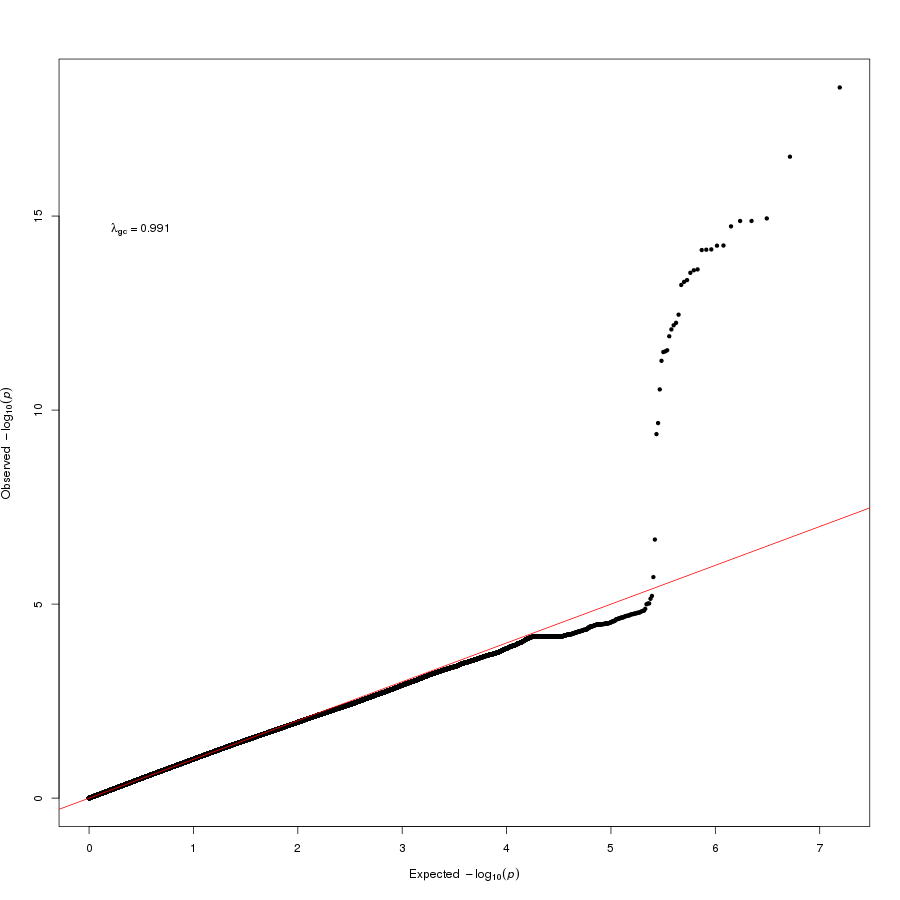


**Supplementary Figure 4B.** Local_AB42_Abnormal


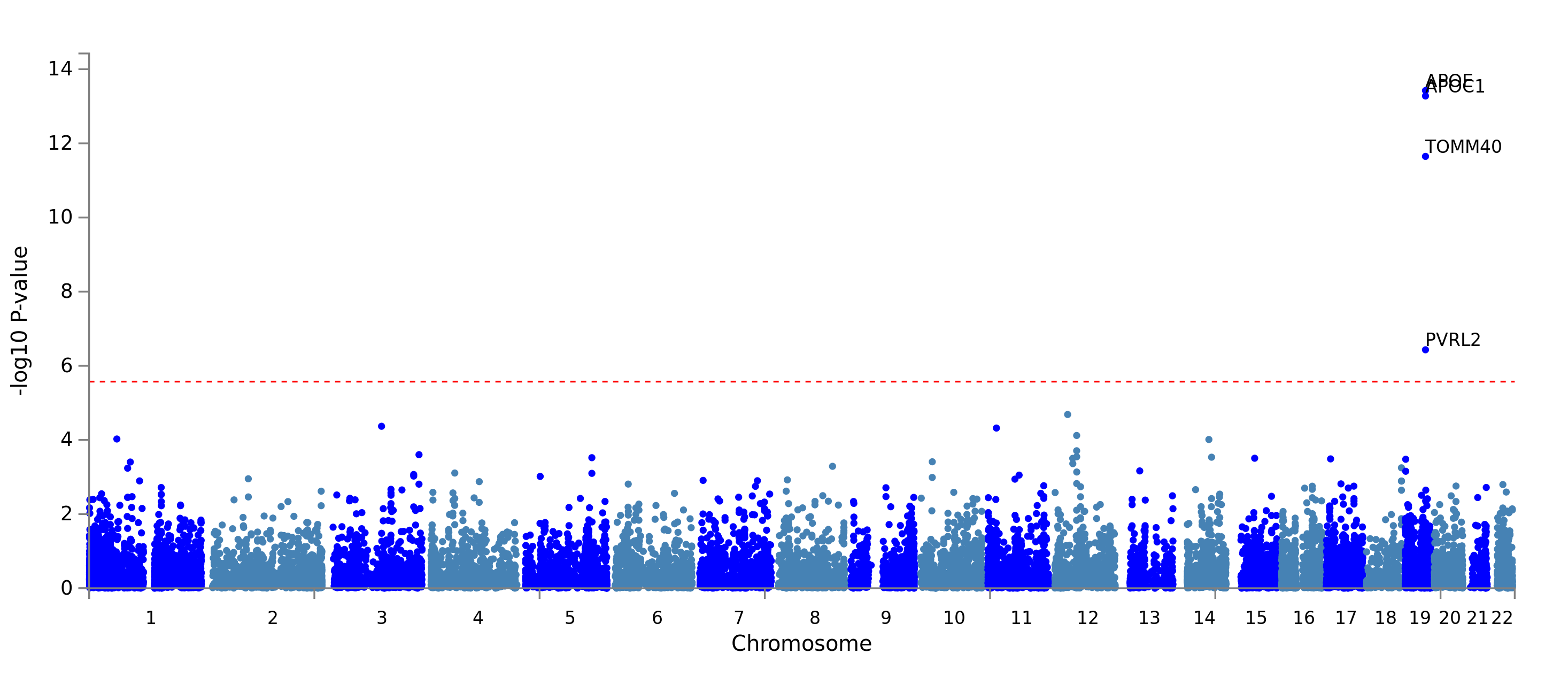


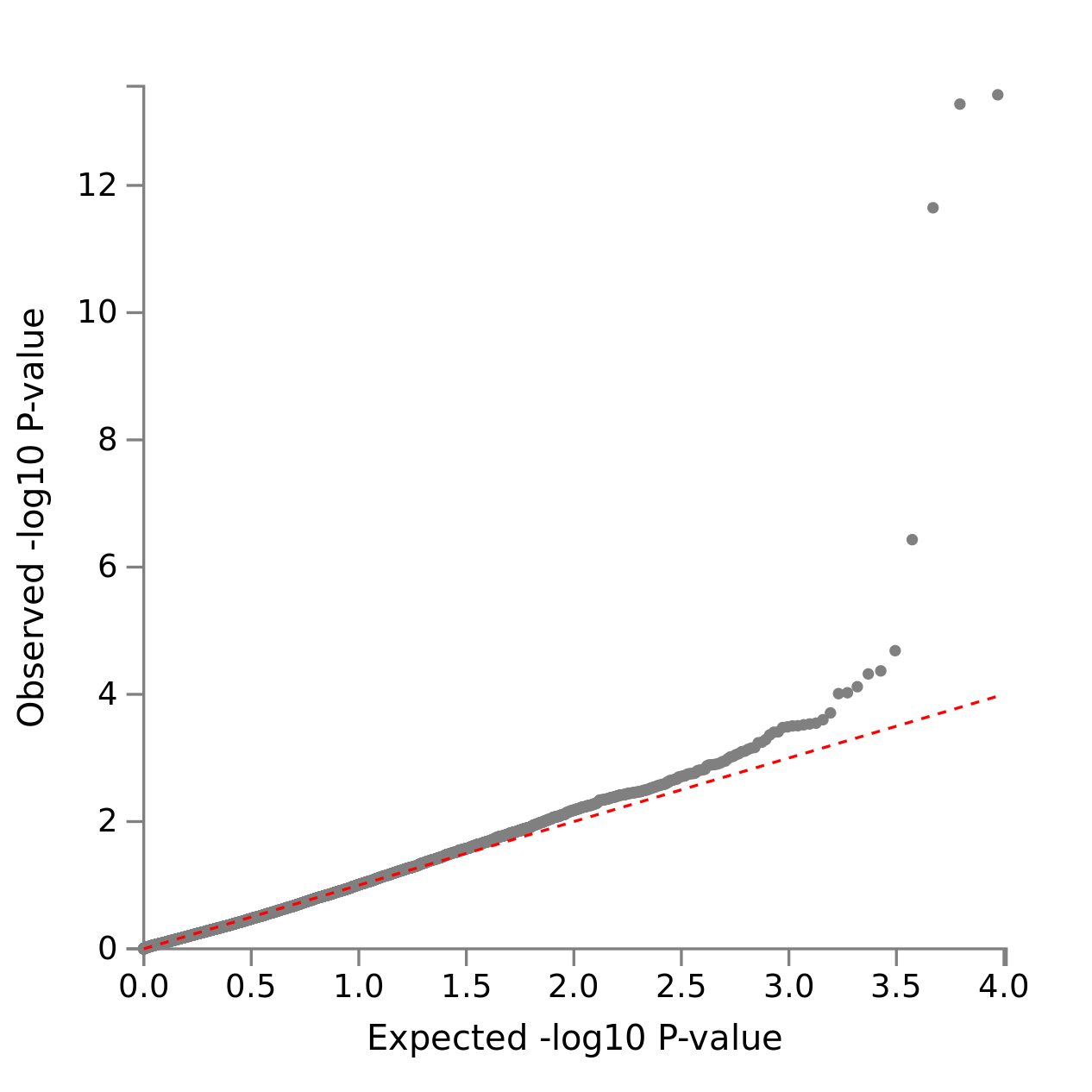


**Supplementary Figure 5A.** AB_Zscore


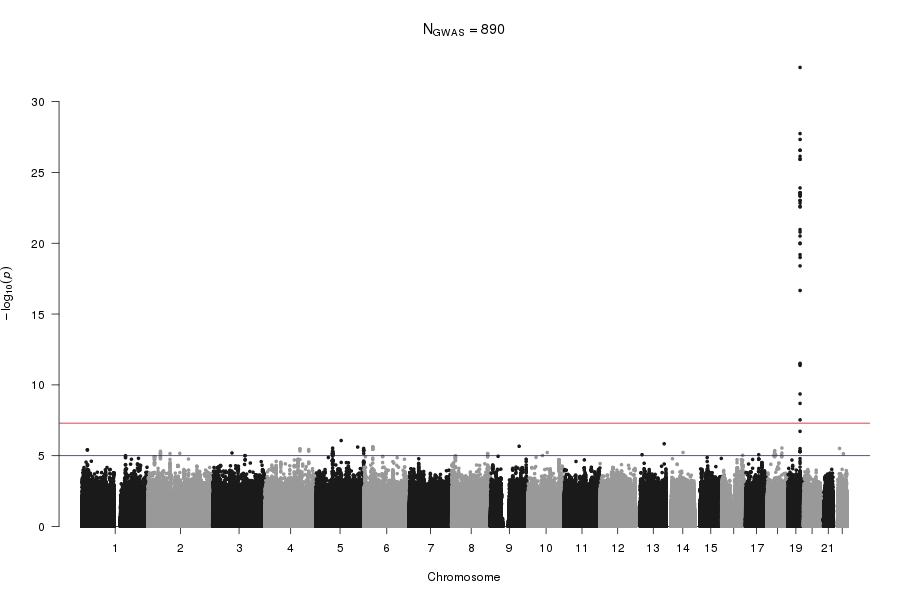


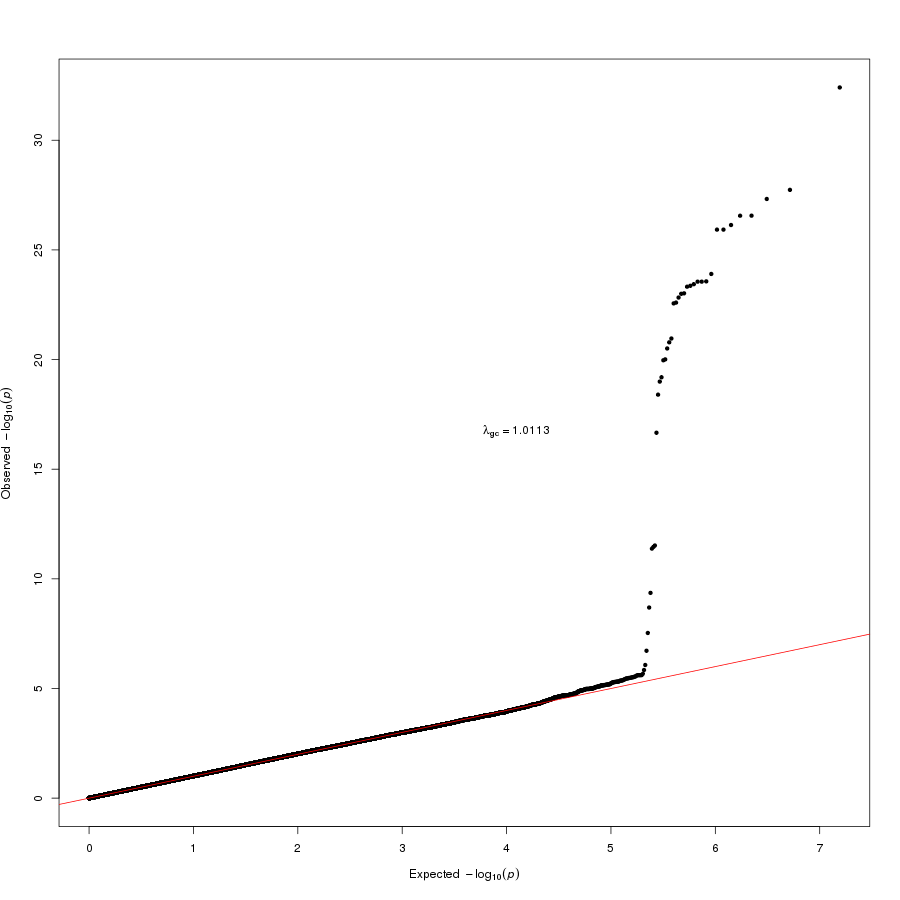


**Supplementary Figure 5B.** AB_Zscore


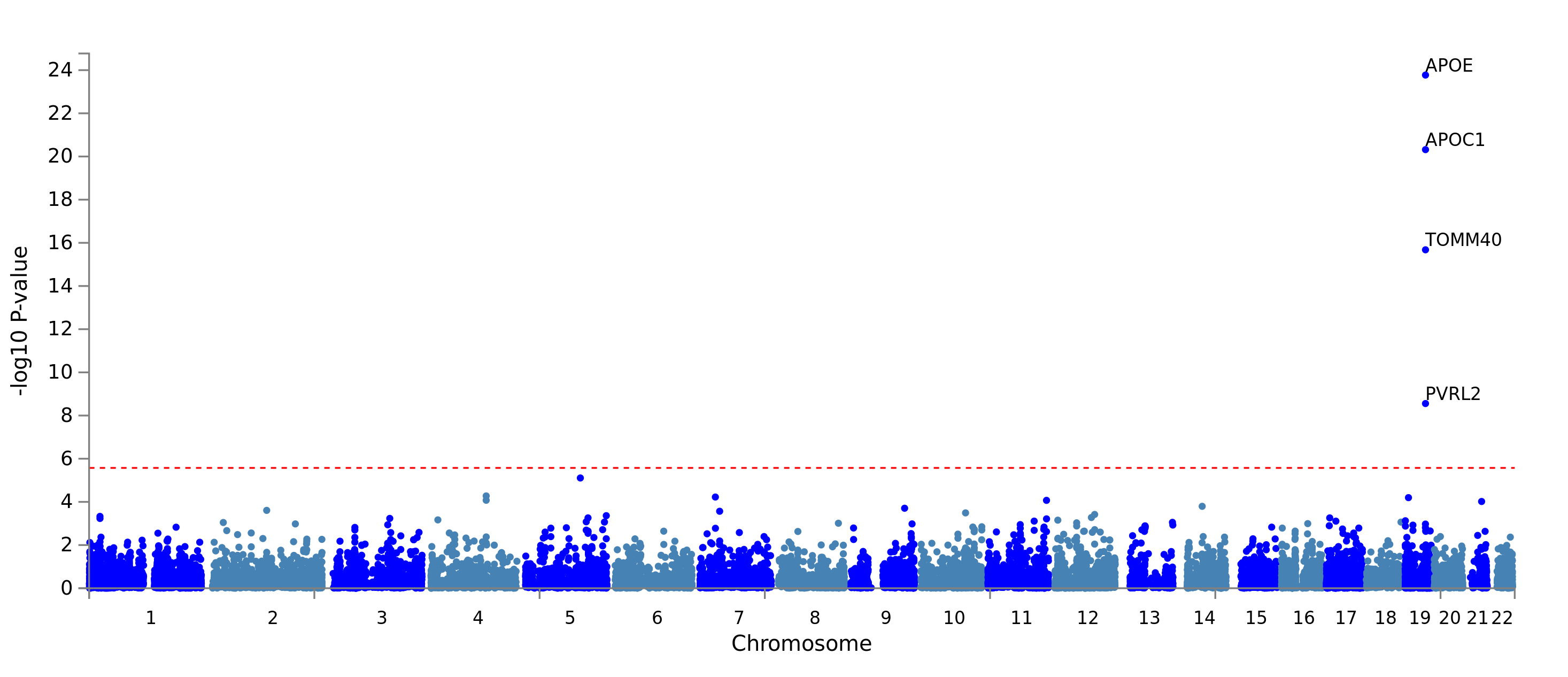


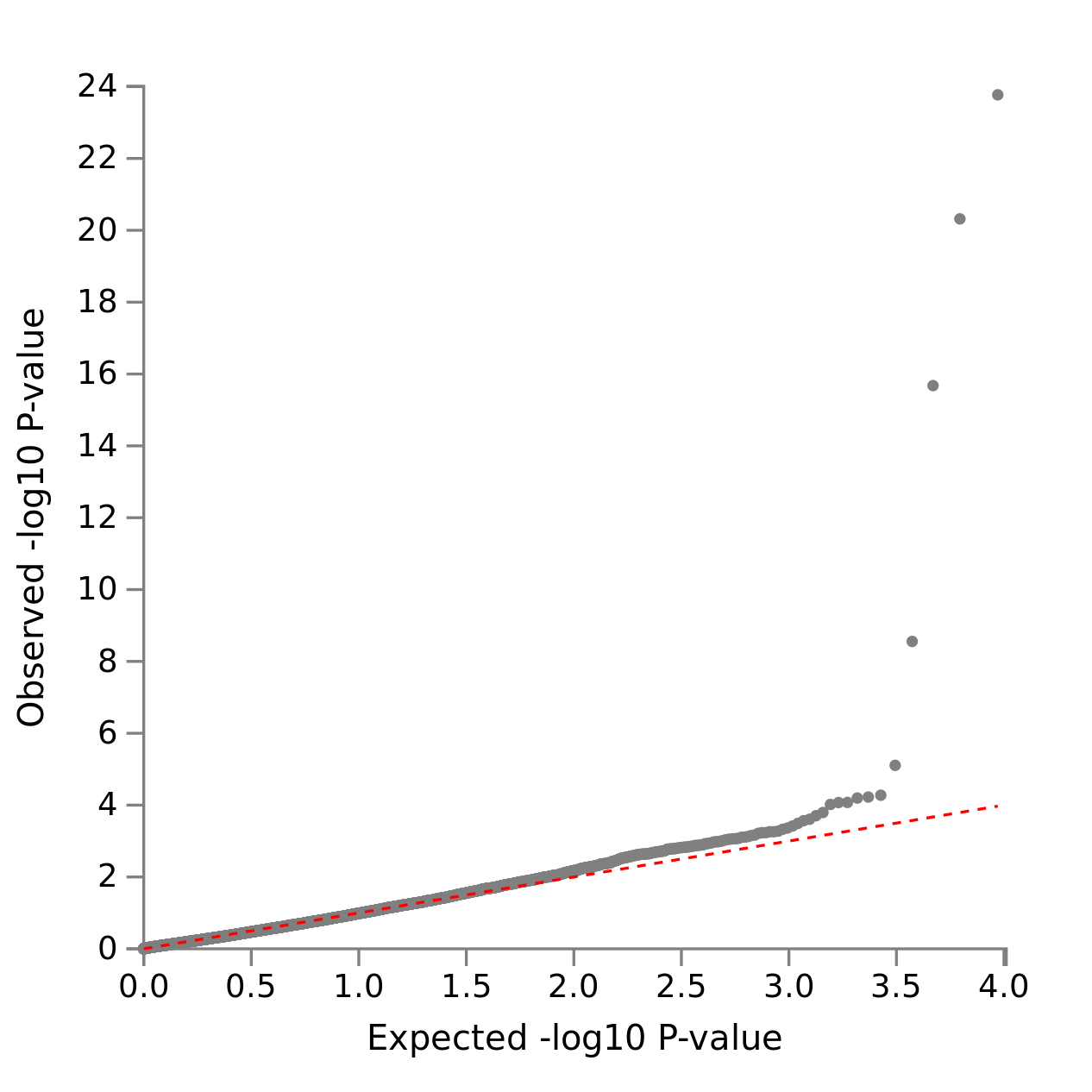


**Supplementary Figure 6A.** log_Central_CSF_AB42


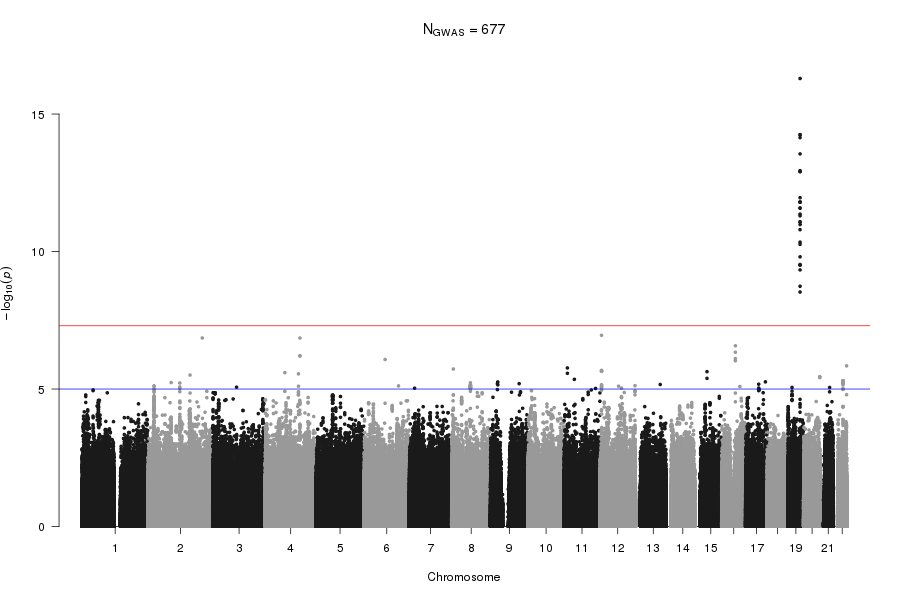

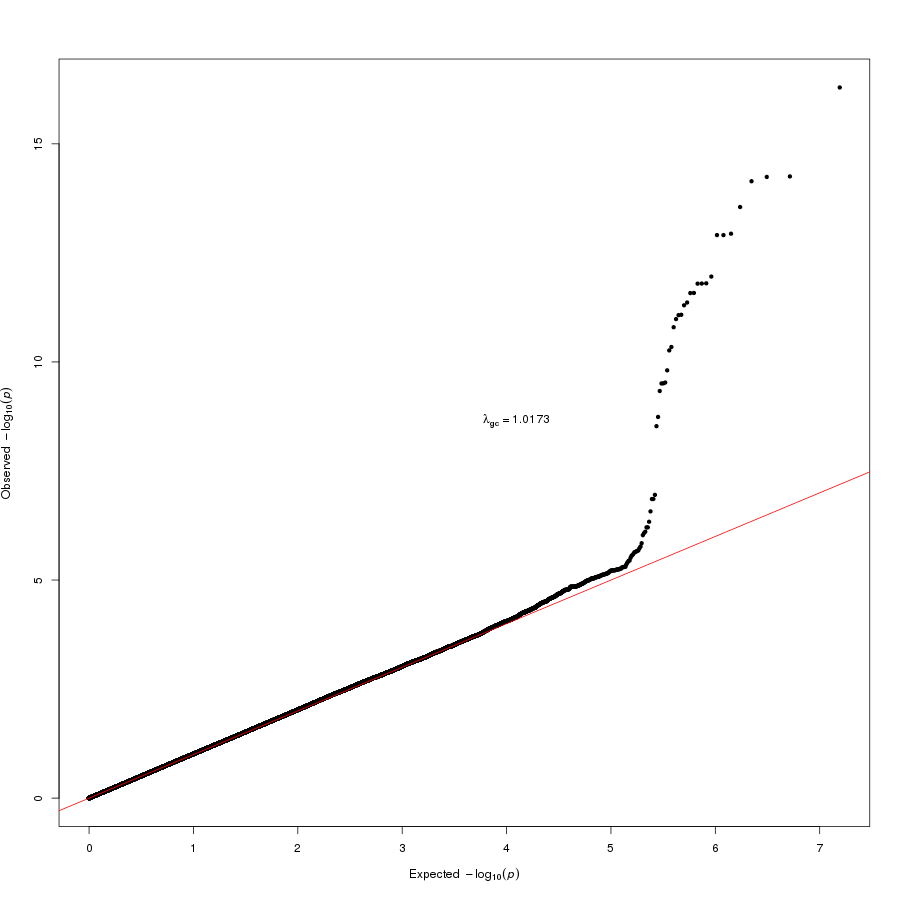


**Supplementary Figure 6B.** log_Central_CSF_AB42


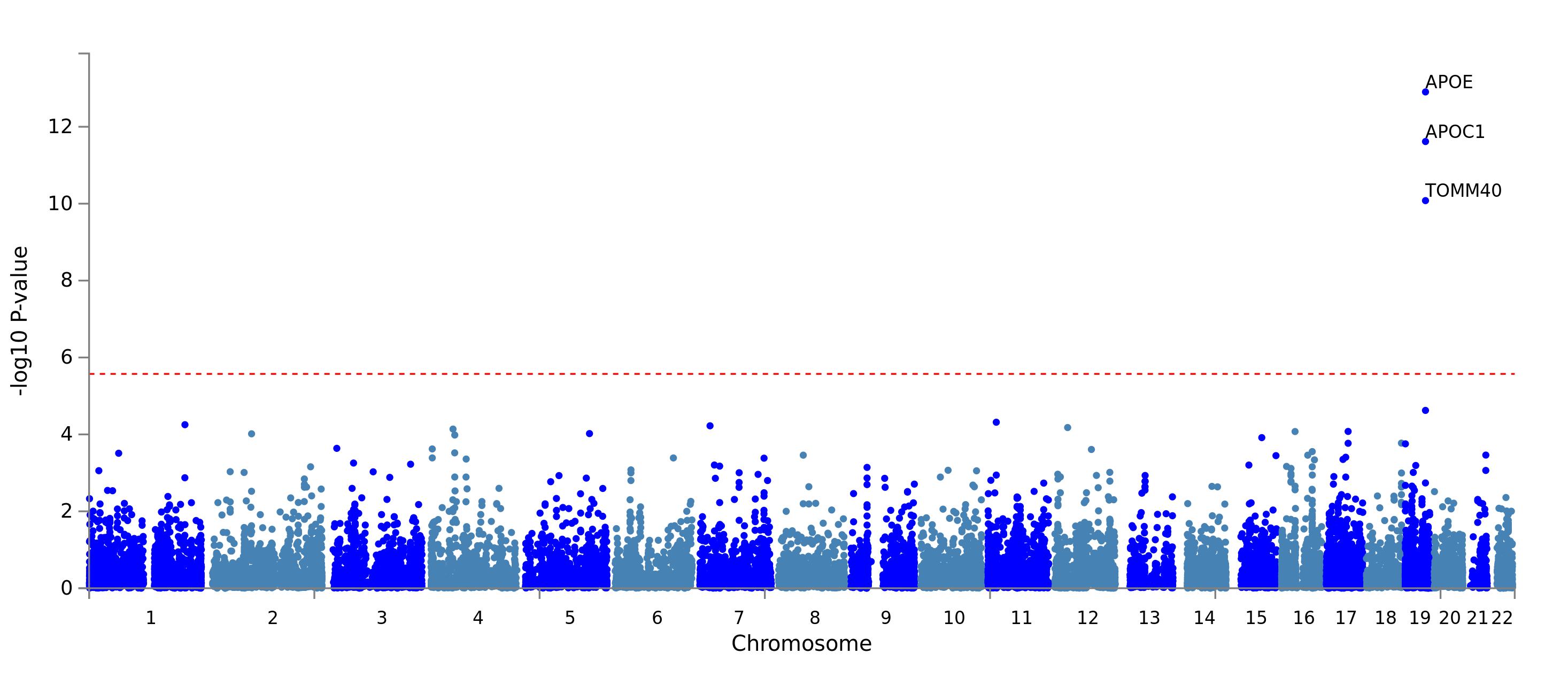


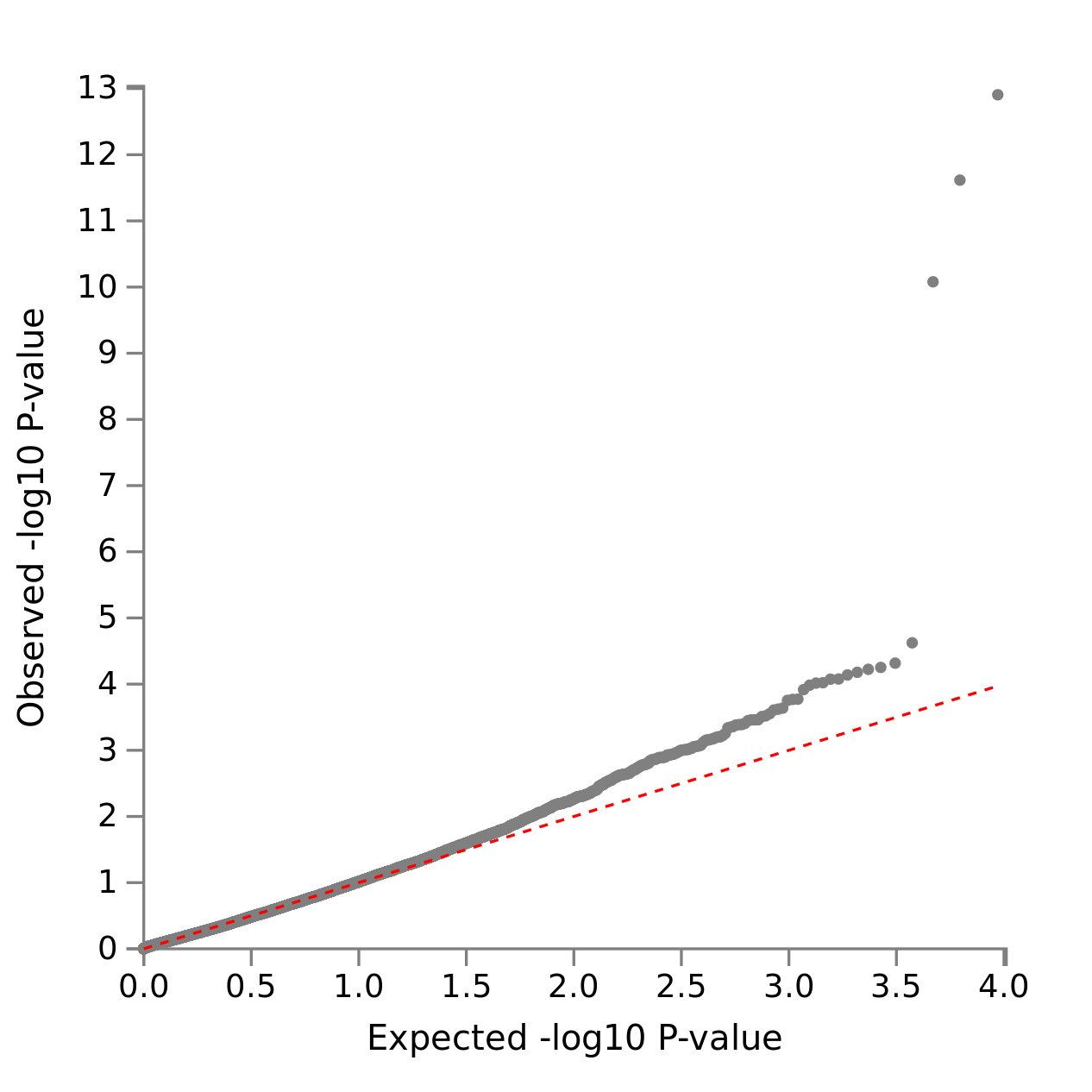


**Supplementary Figure 7A.** log_Central_CSF_AB4240ratio


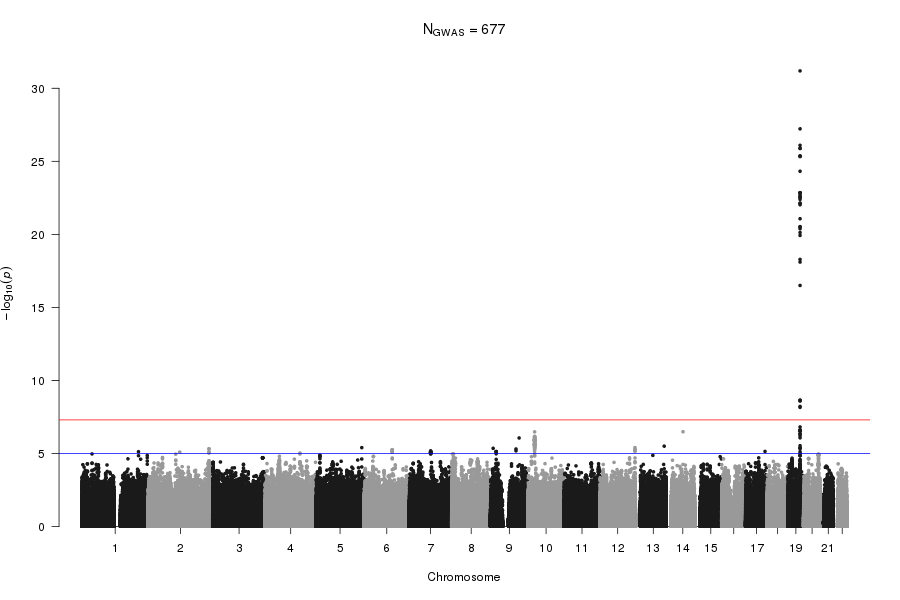


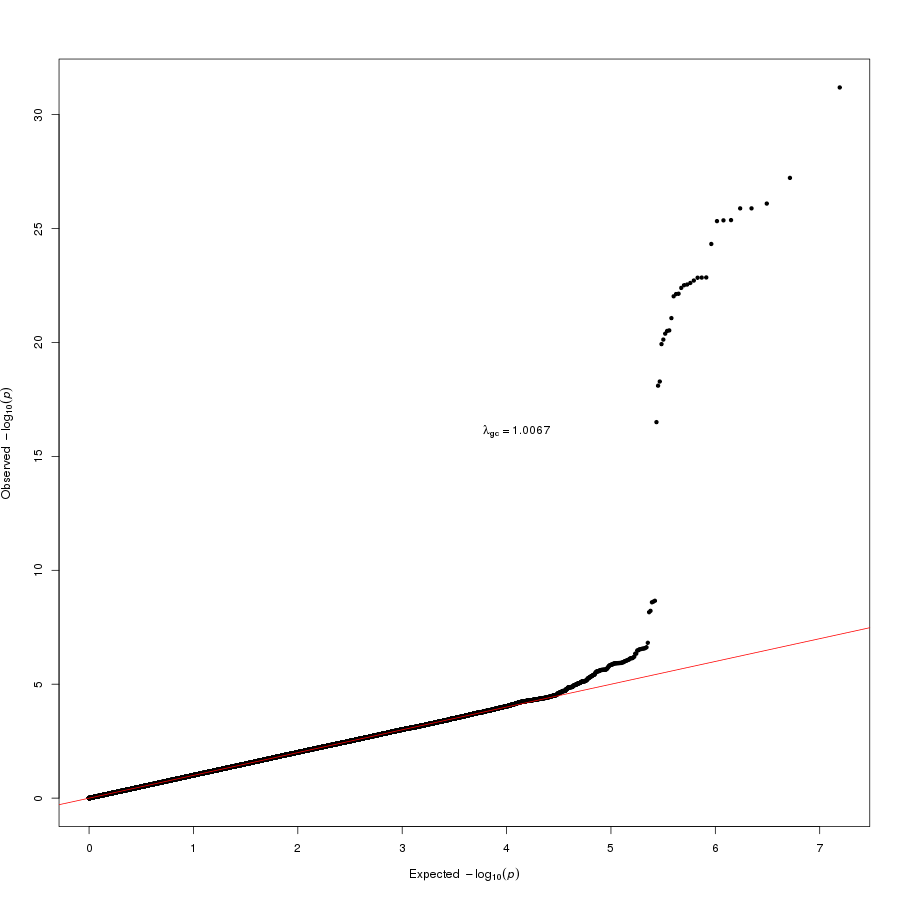


**Supplementary Figure 7B.** log_Central_CSF_AB4240ratio


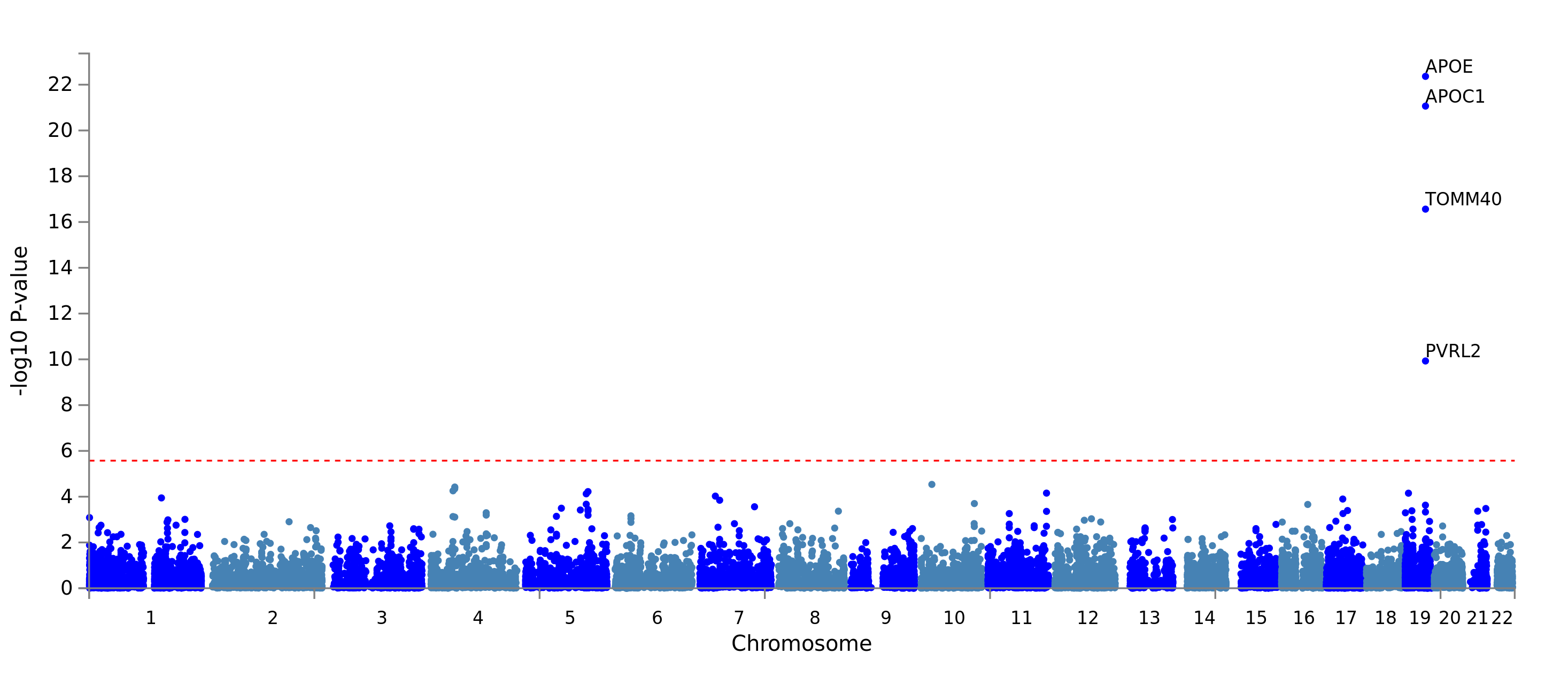


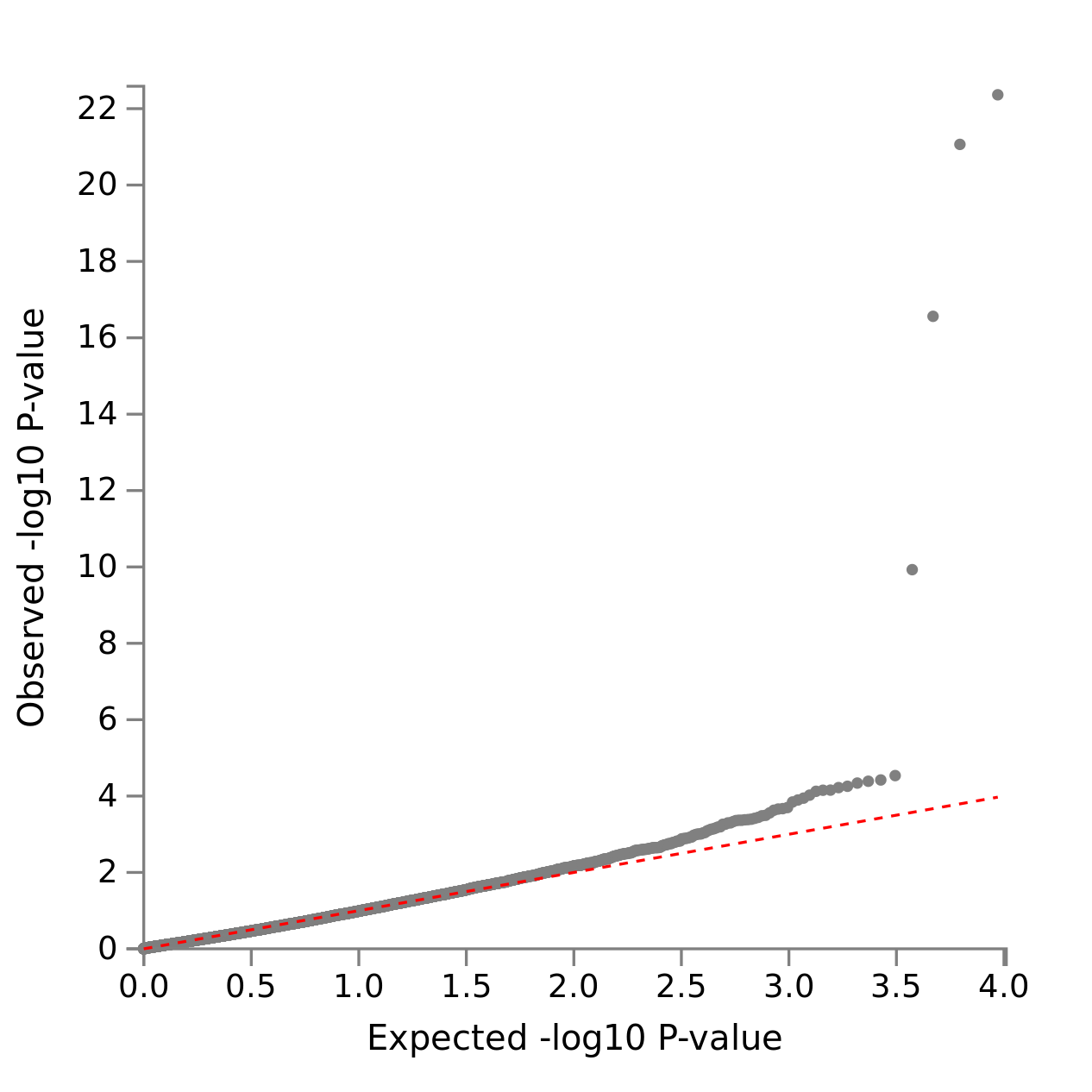


**Supplementary Figure 8A.** CSF-Ab38


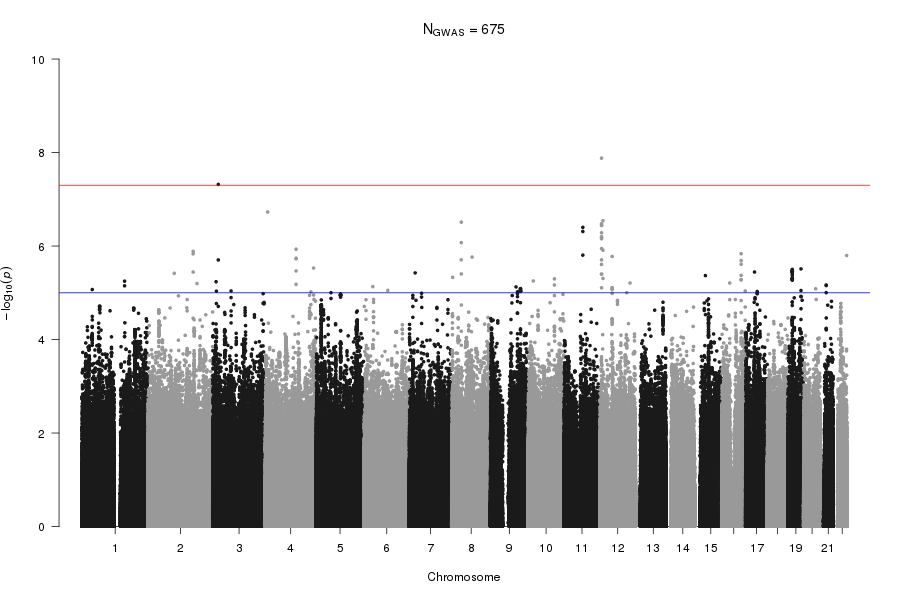


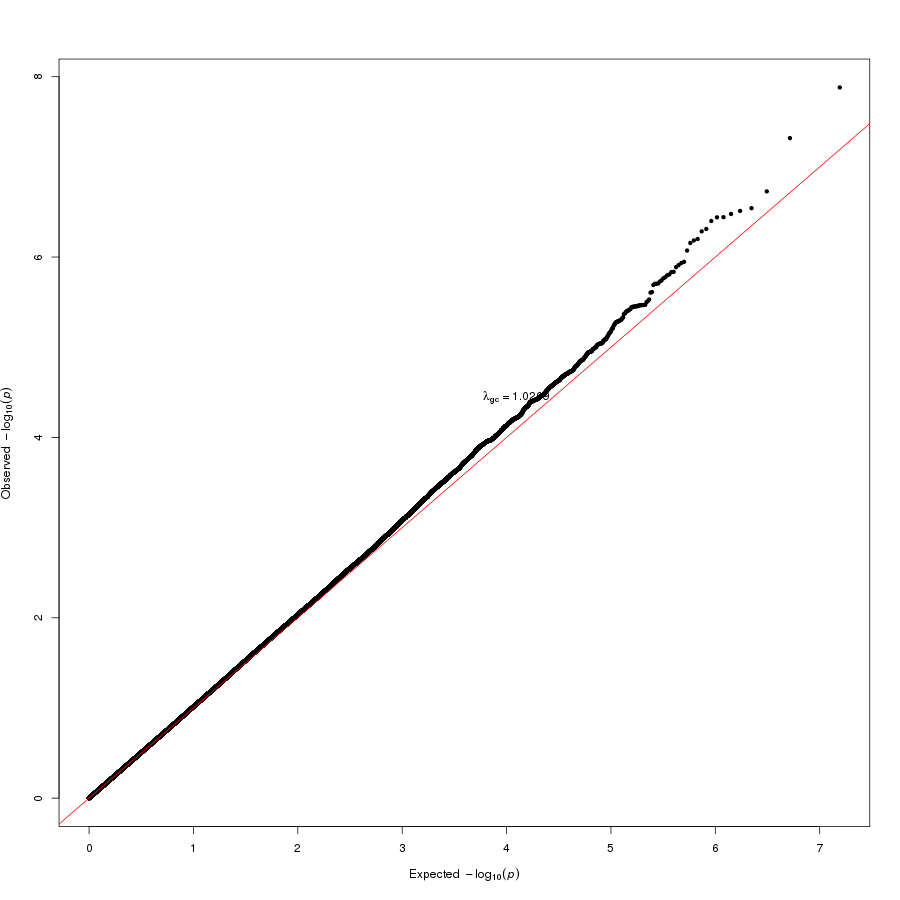


**Supplementary Figure 8B.** CSF-Ab38


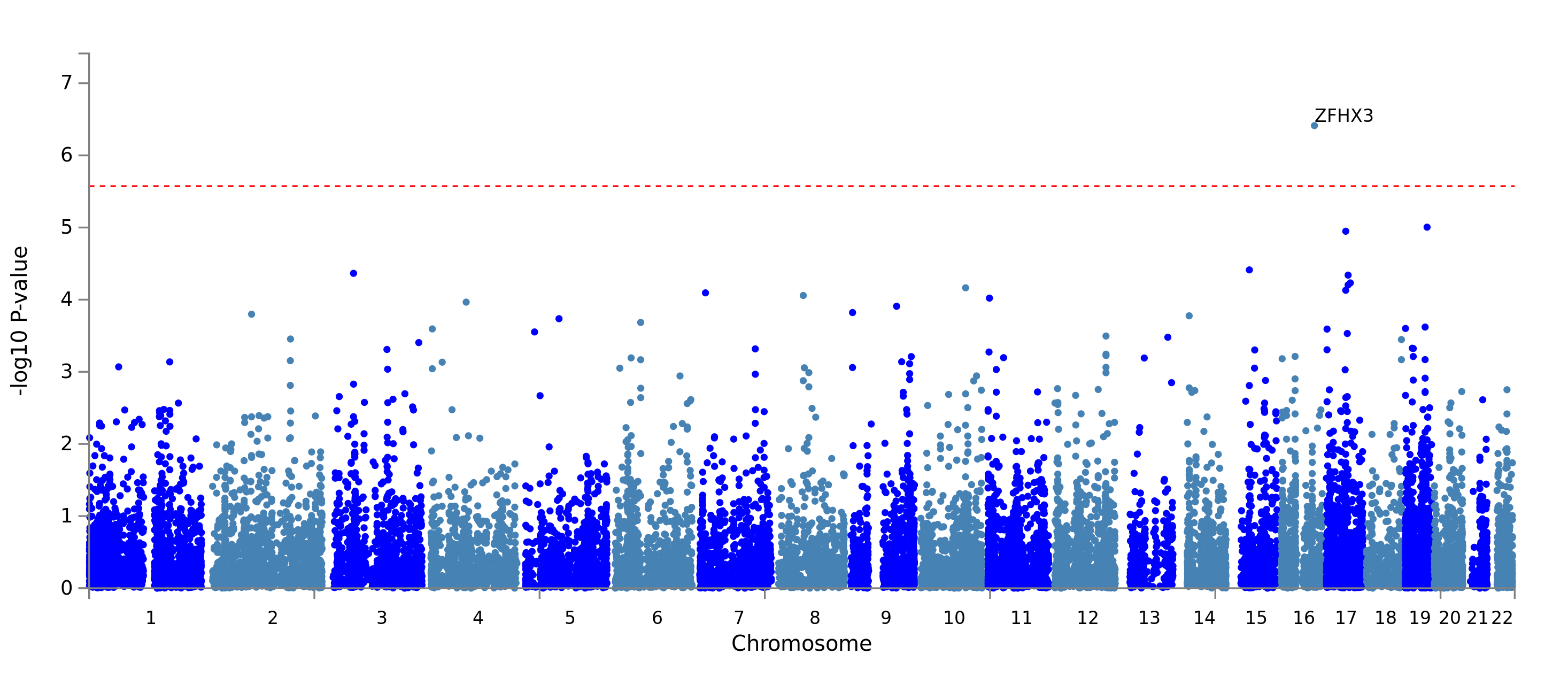


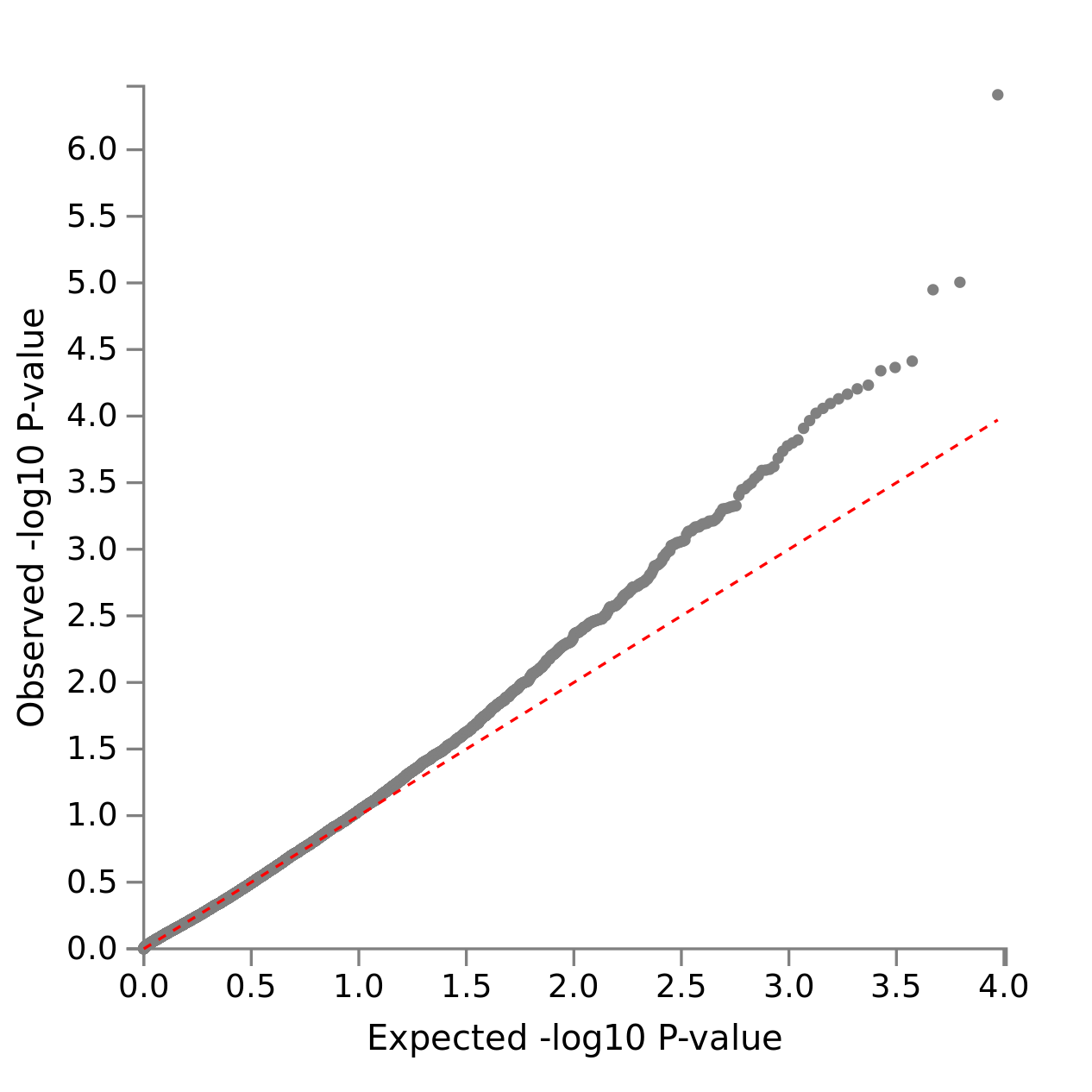


**Supplementary Figure 9A.** CSF-Ab40


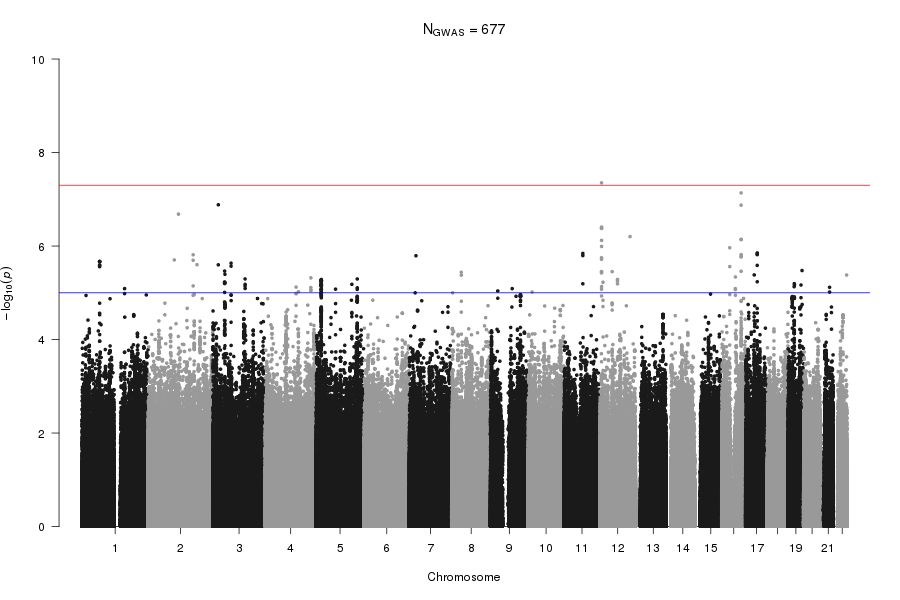

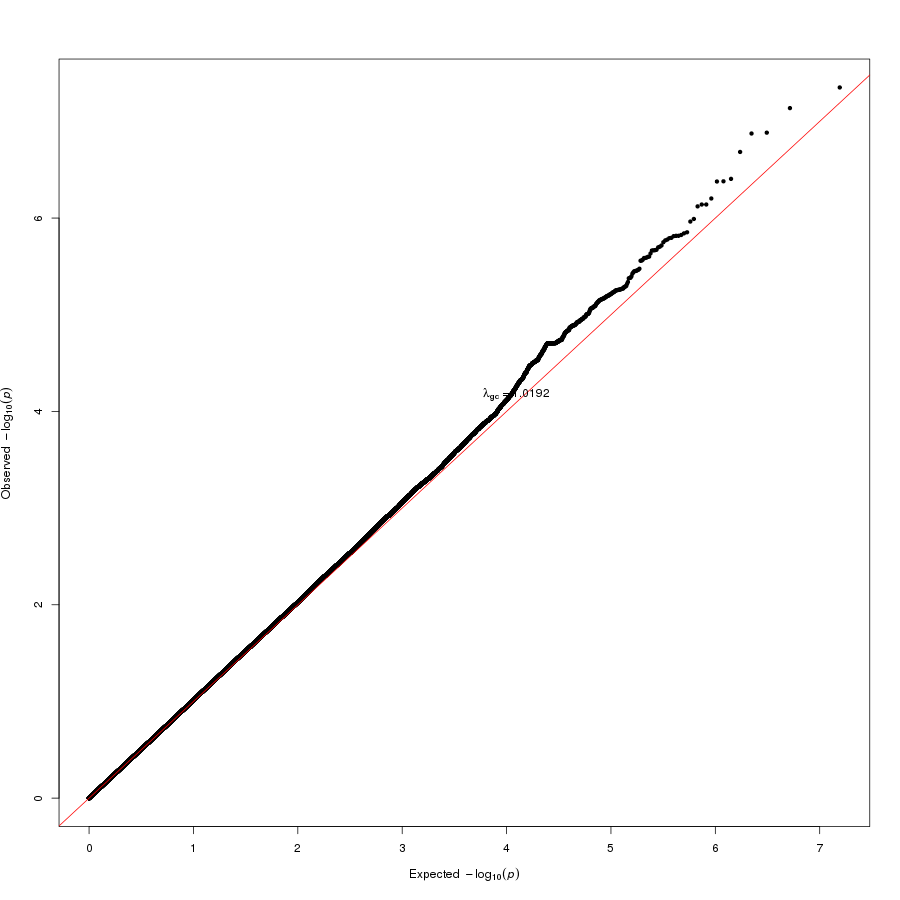


**Supplementary Figure 9B.** CSF-Ab40


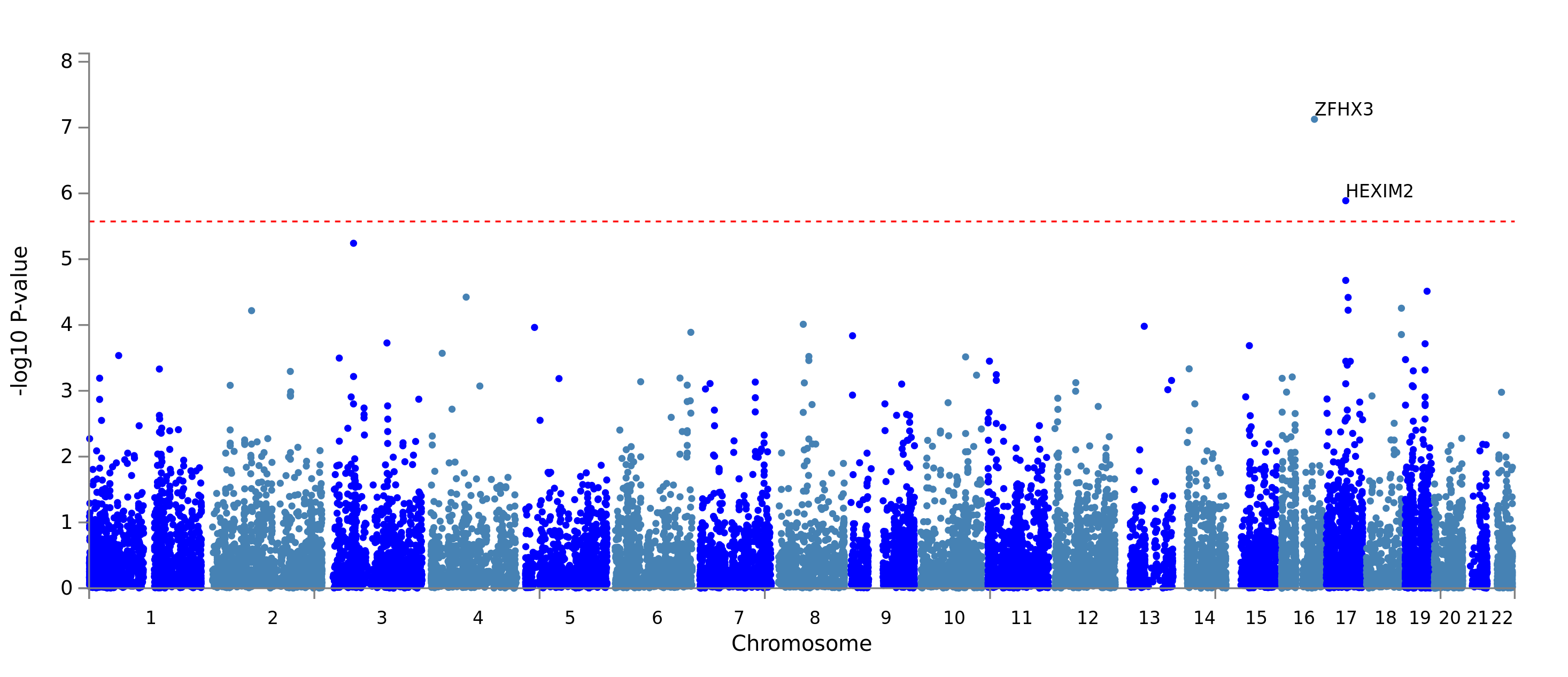


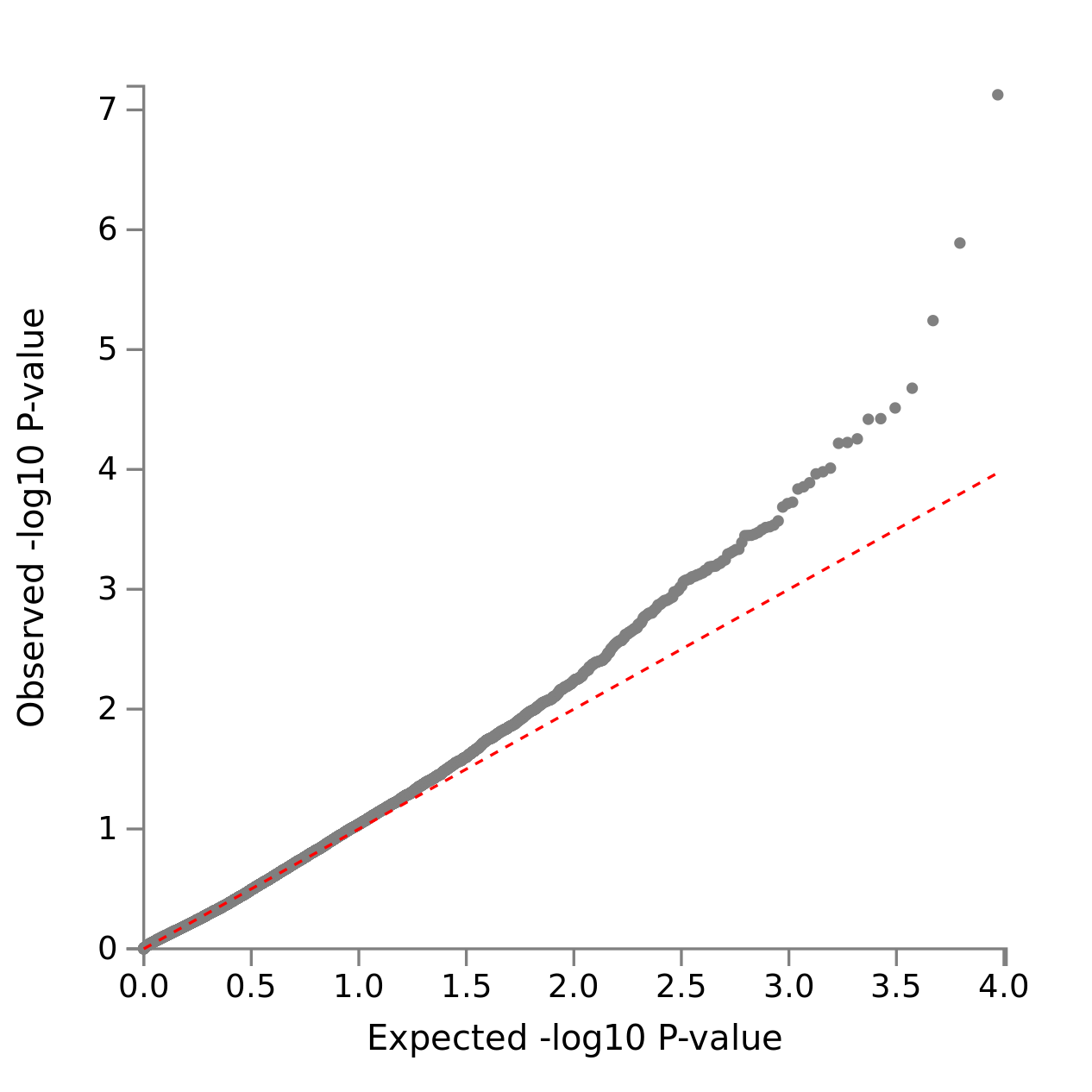


**Supplementary Figure 10A.** Local_TTAU_Abnormal


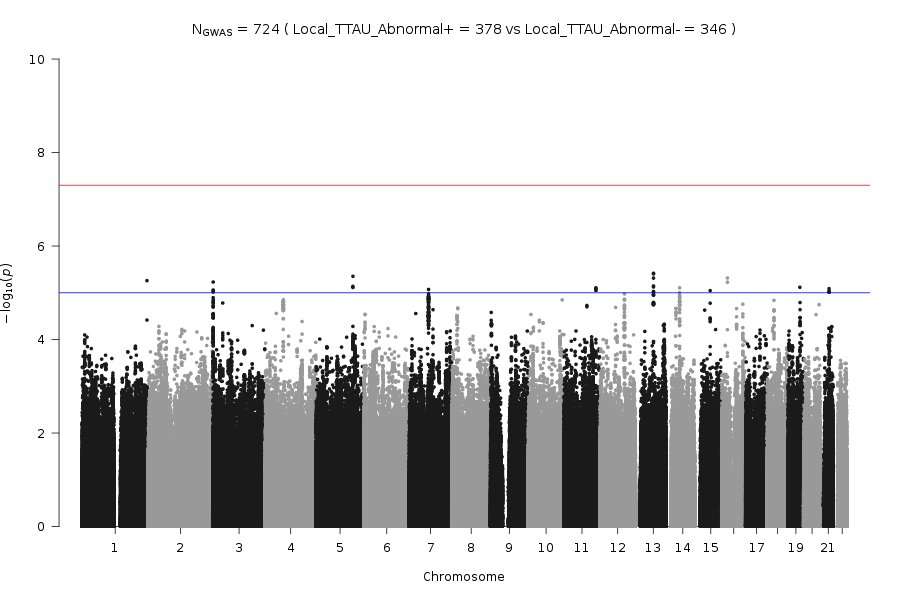

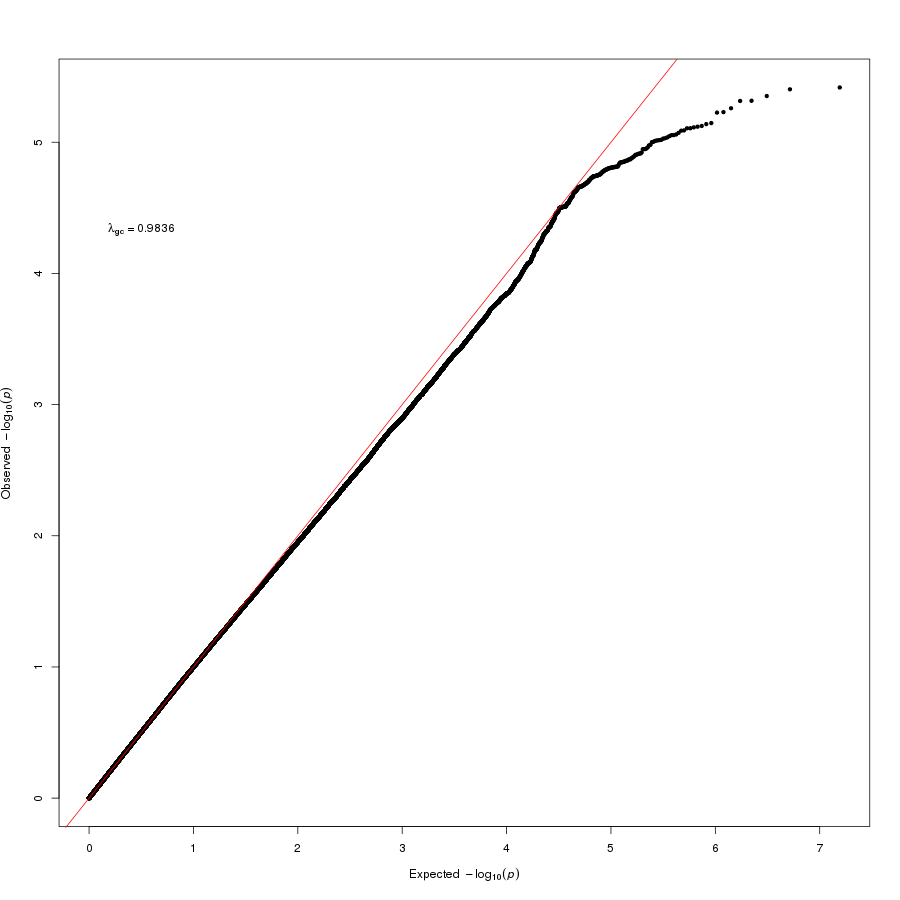


**Supplementary Figure 10B.** Local_TTAU_Abnormal


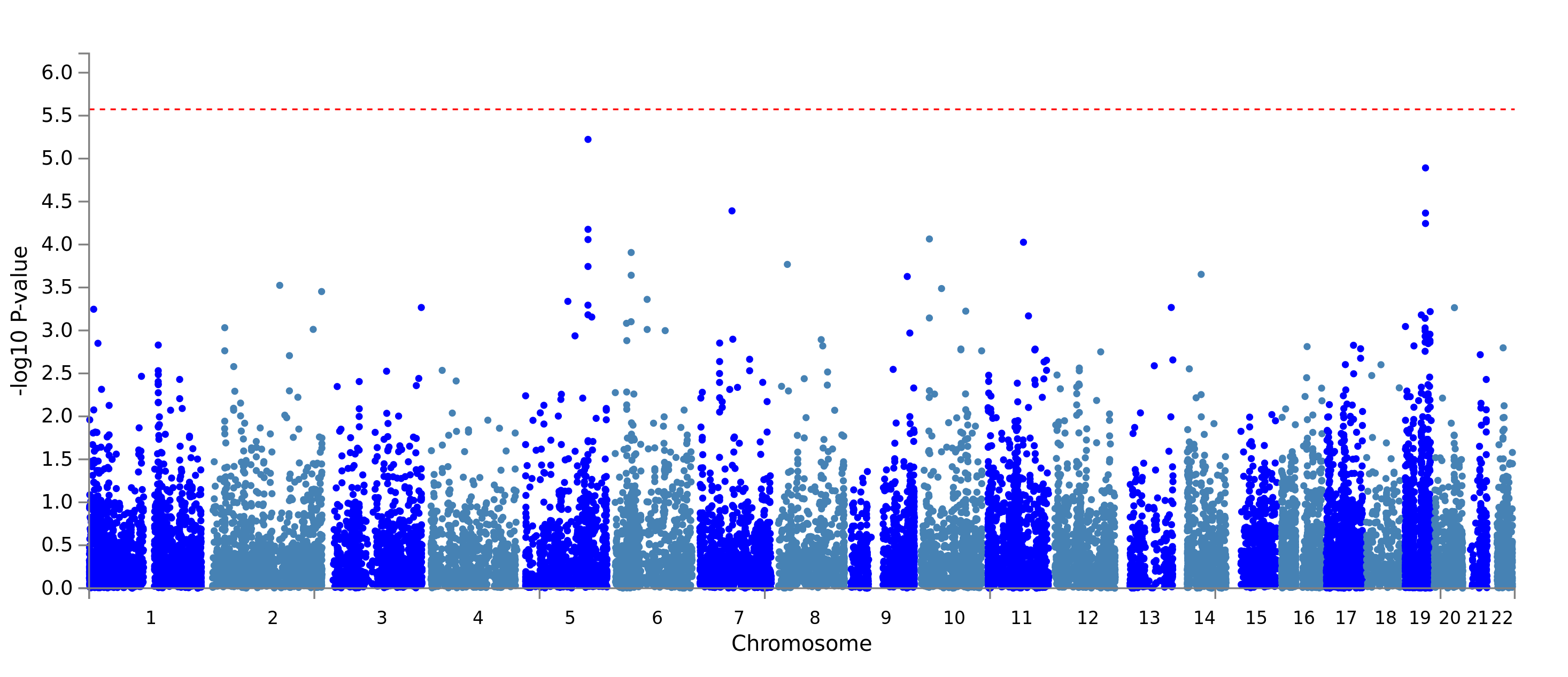


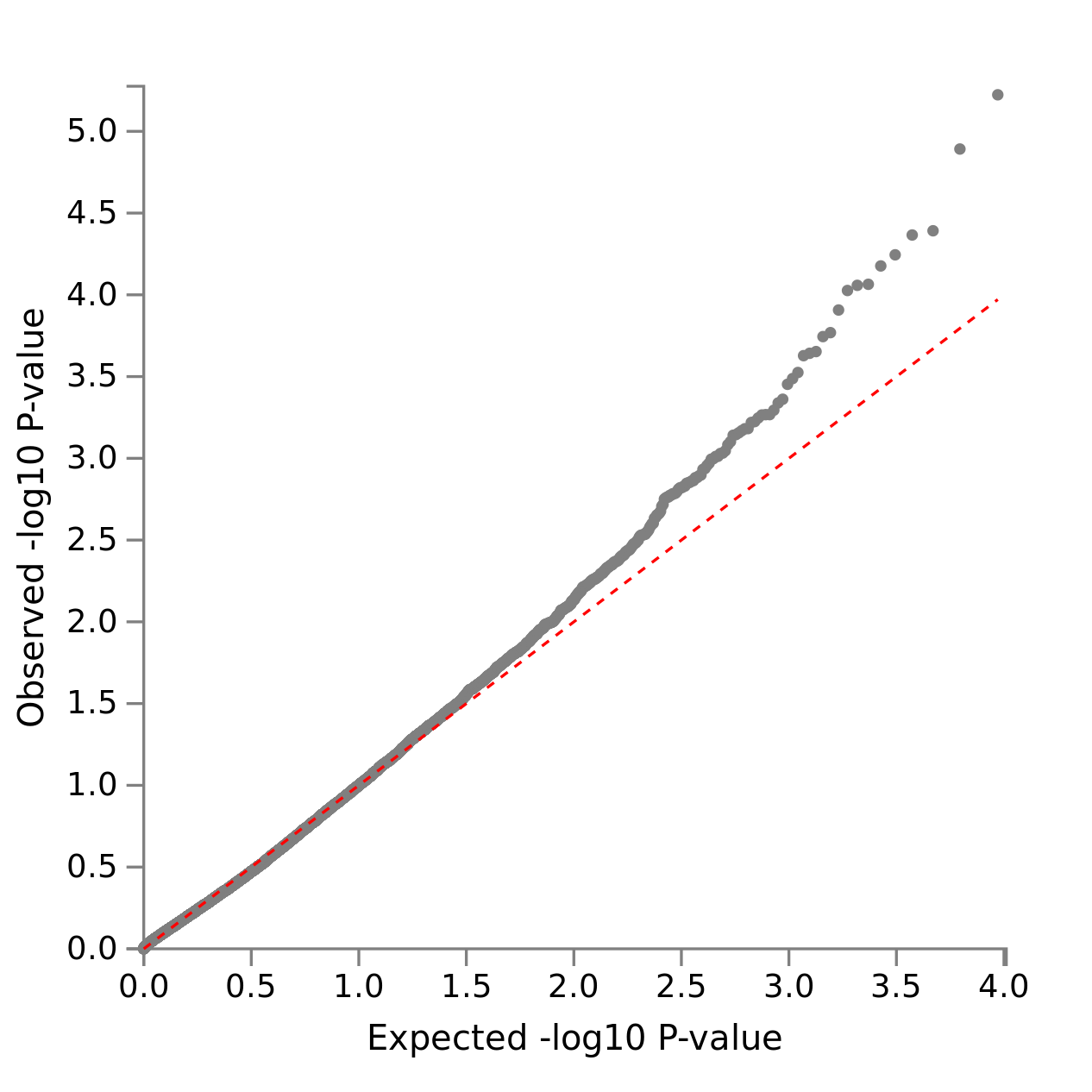


**Supplementary Figure 11A.** Ttau_ASSAY_Zscore


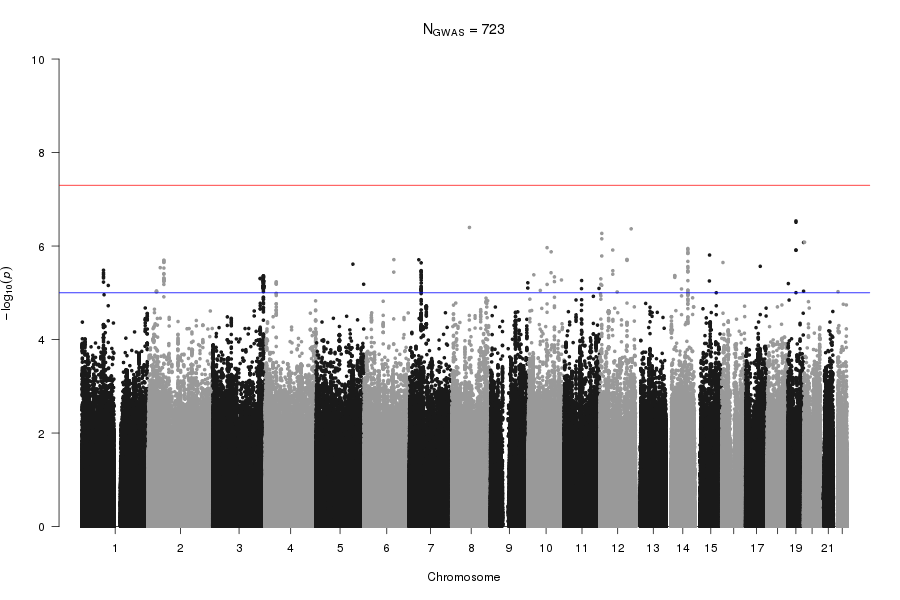

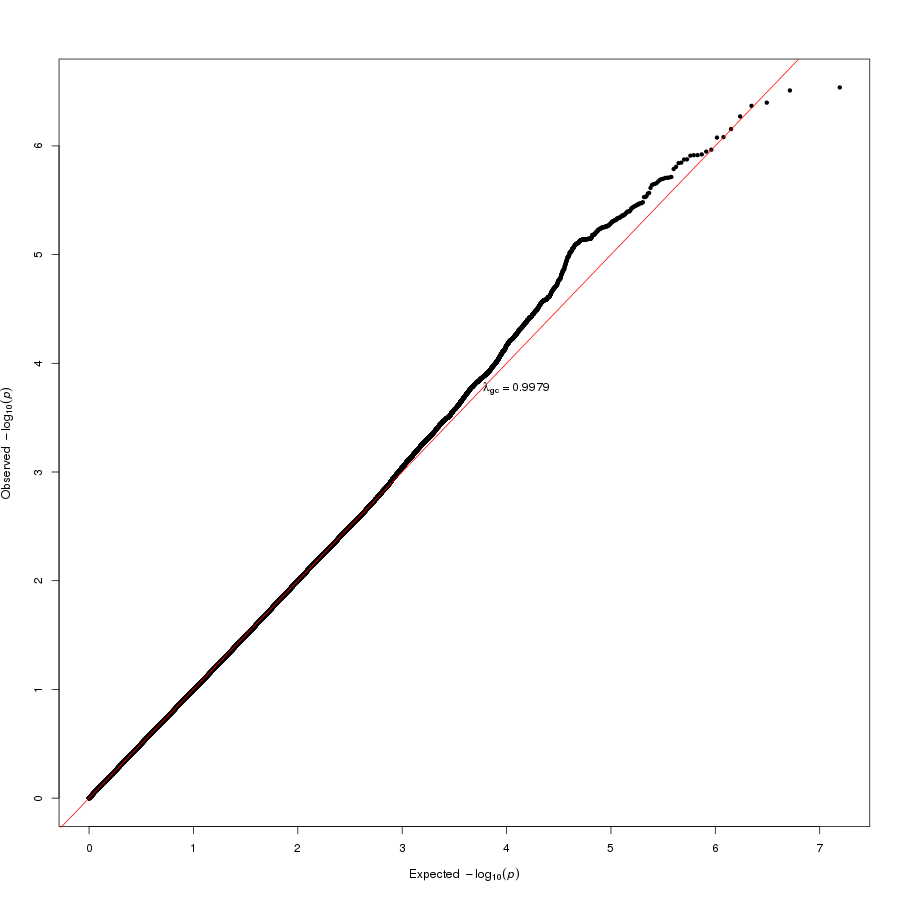


**Supplementary Figure 11B.** Ttau_ASSAY_Zscore


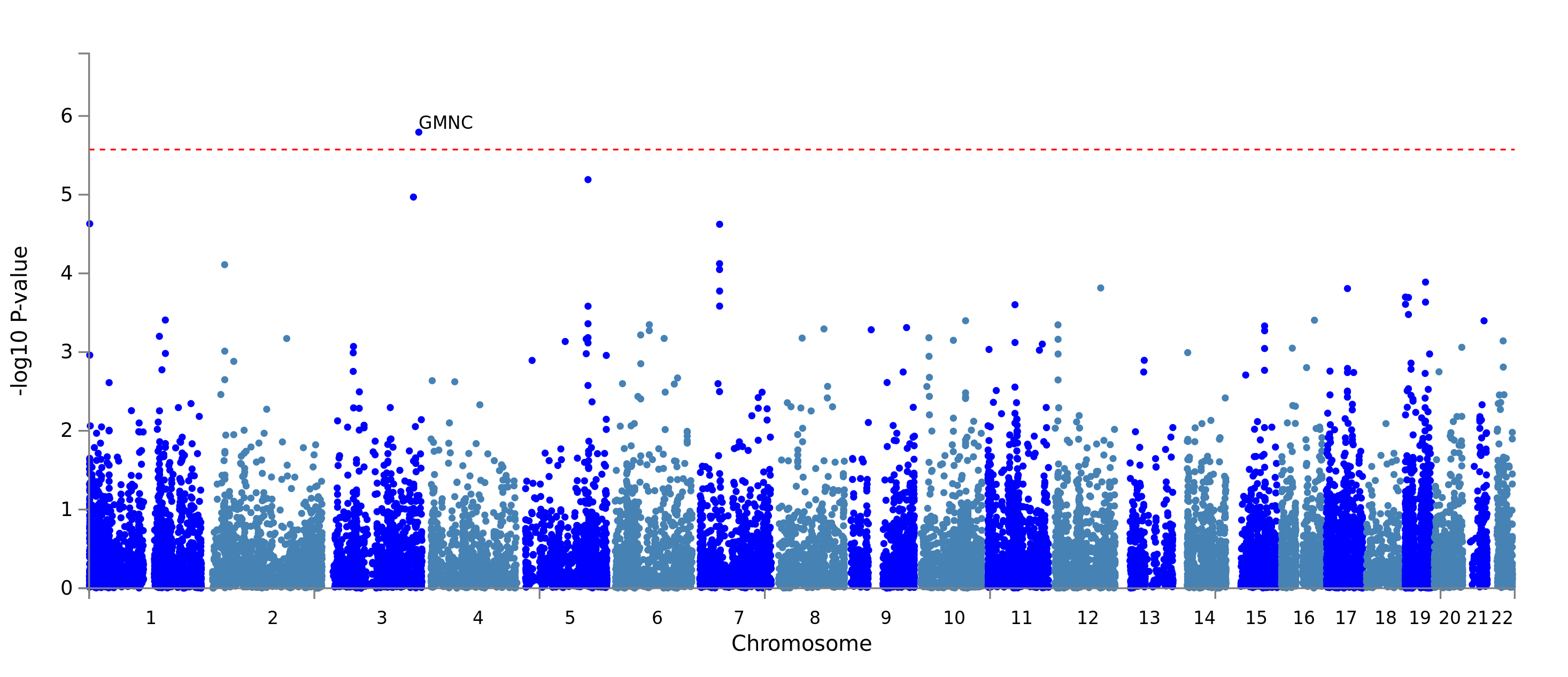


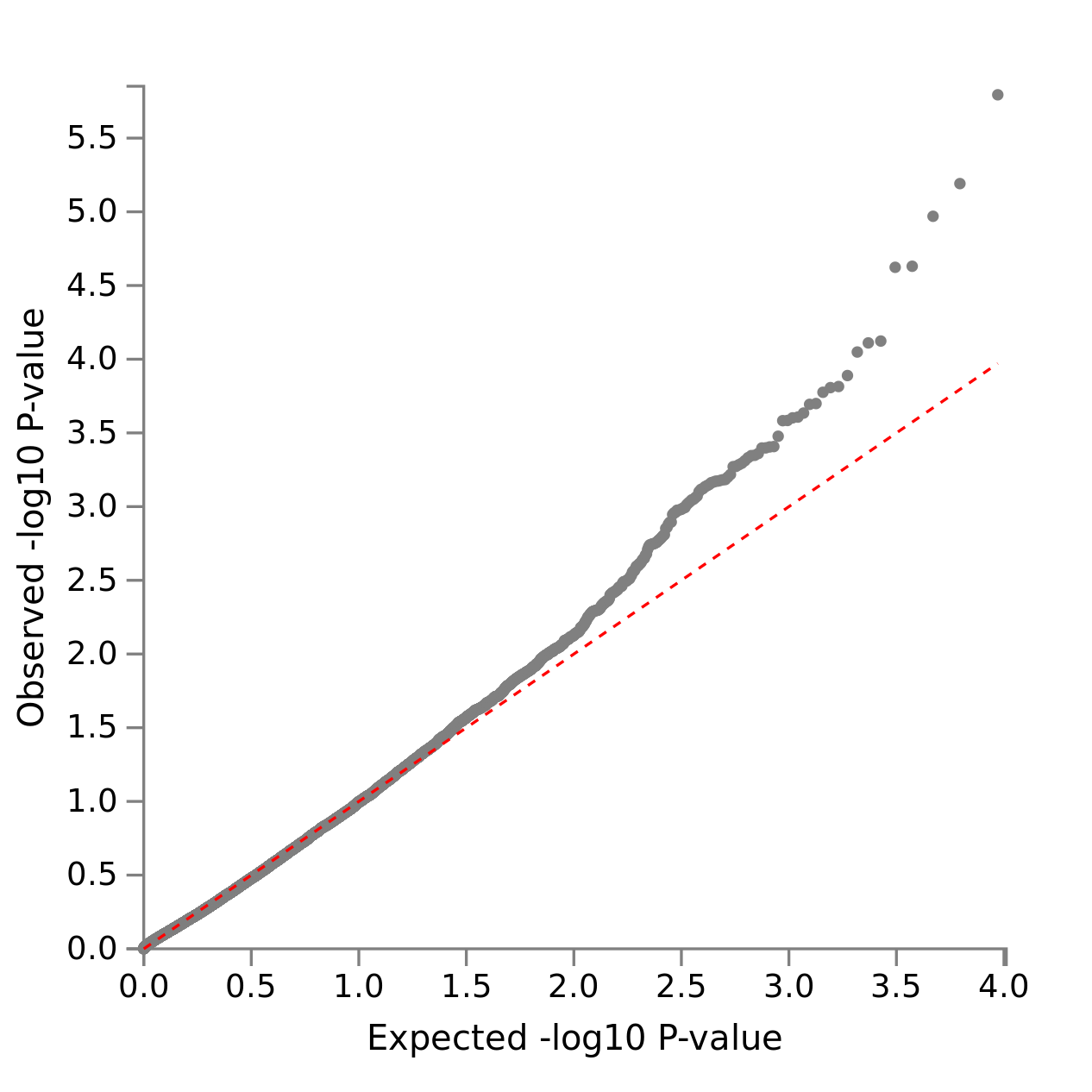


**Supplementary Figure 12A.** Local_PTAU_Abnormal


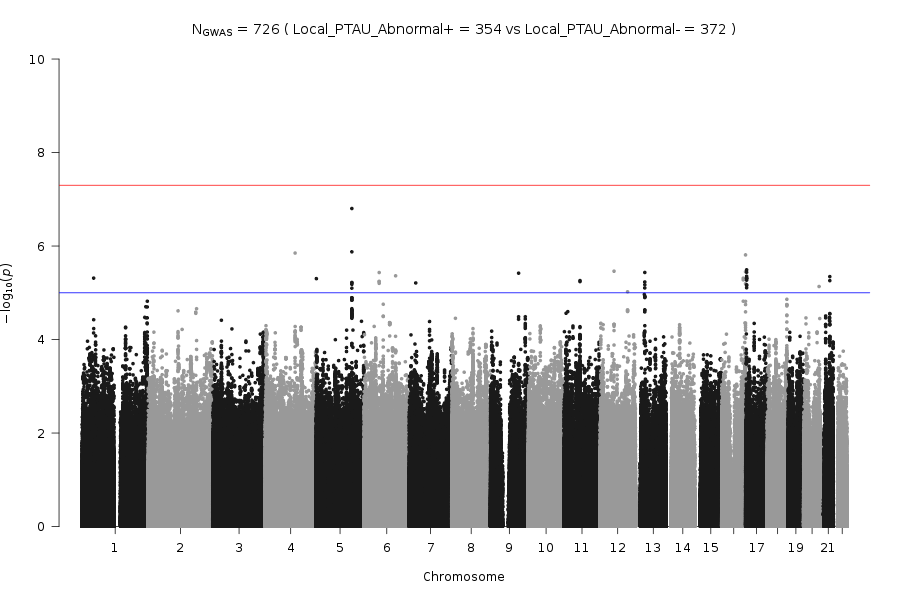

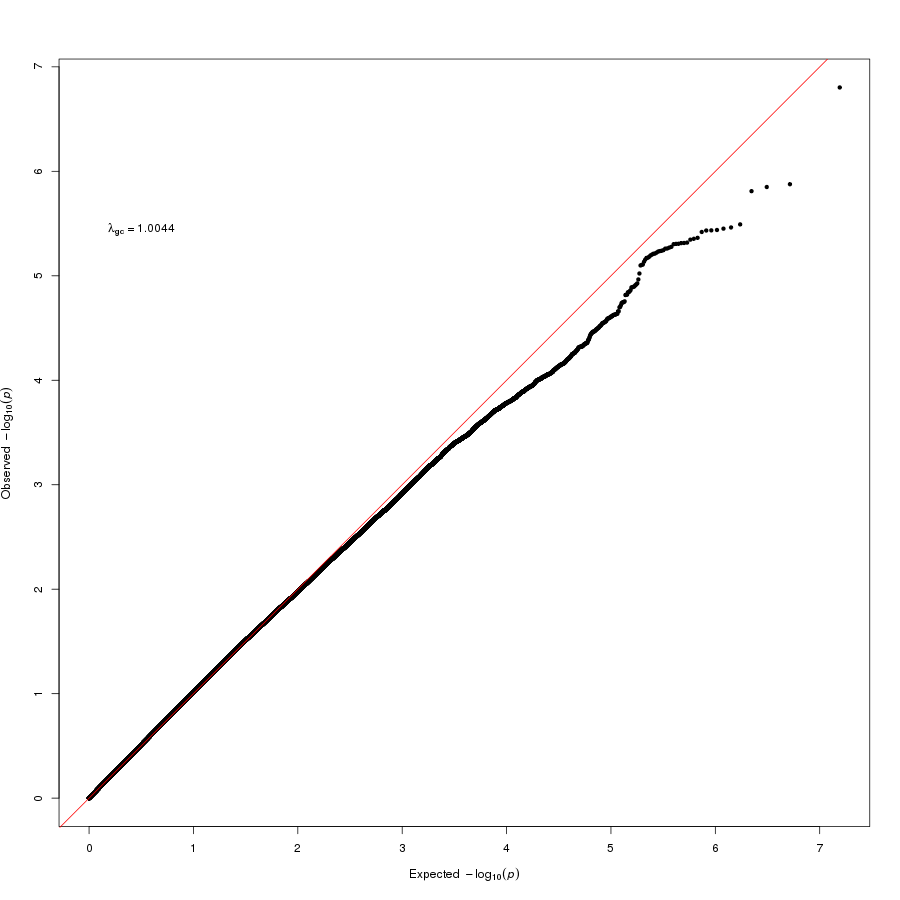


**Supplementary Figure 12B.** Local_PTAU_Abnormal


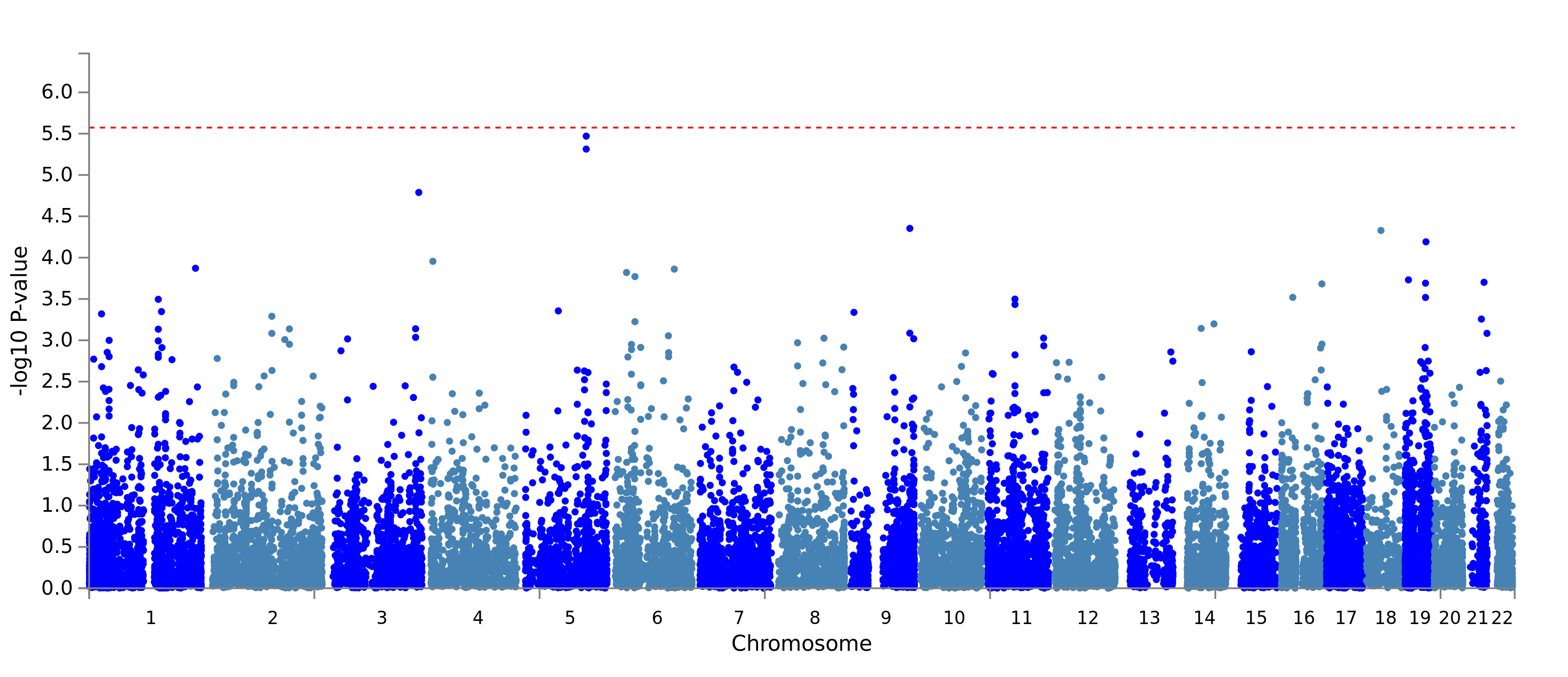


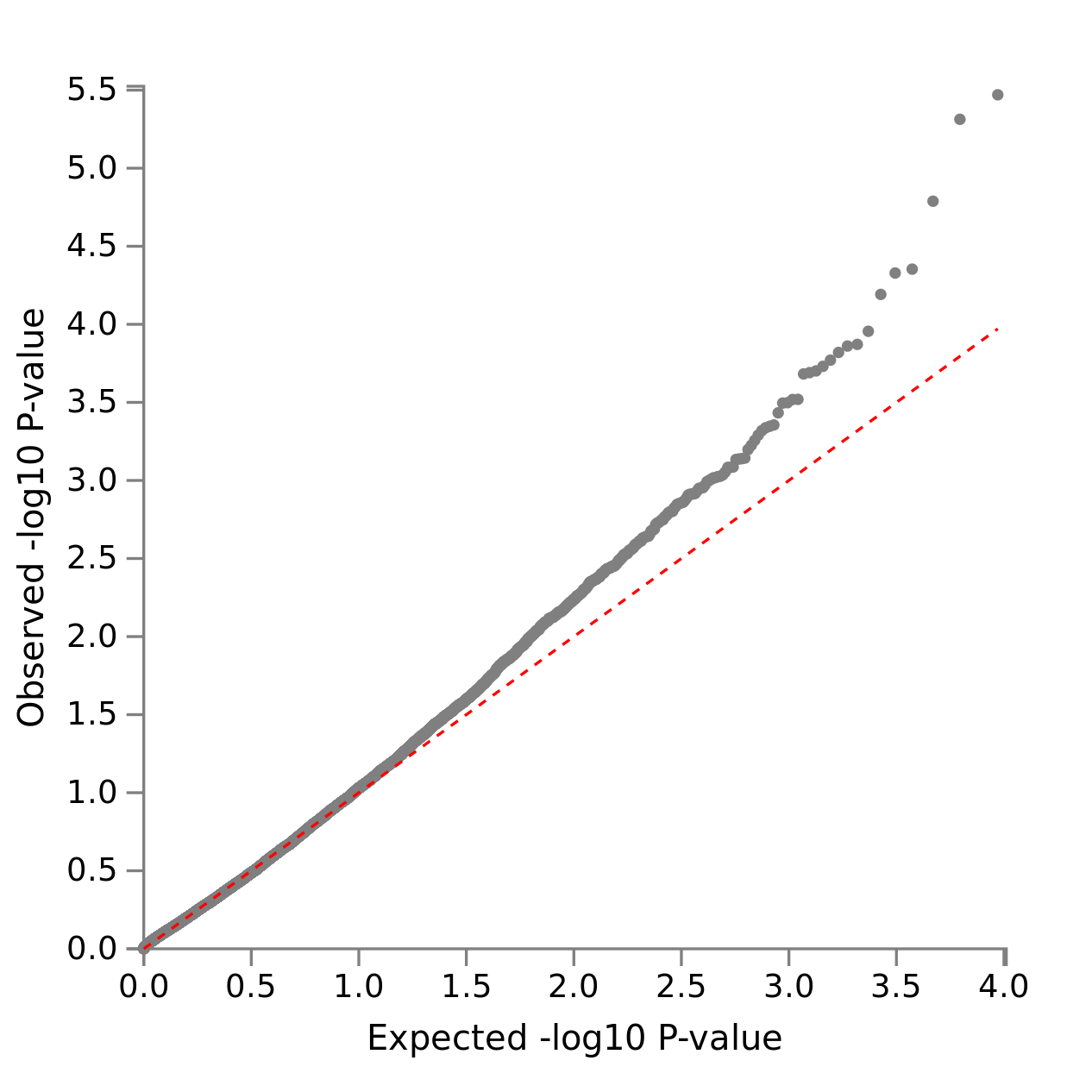


**Supplementary Figure 13A.** Ptau_ASSAY_Zscore


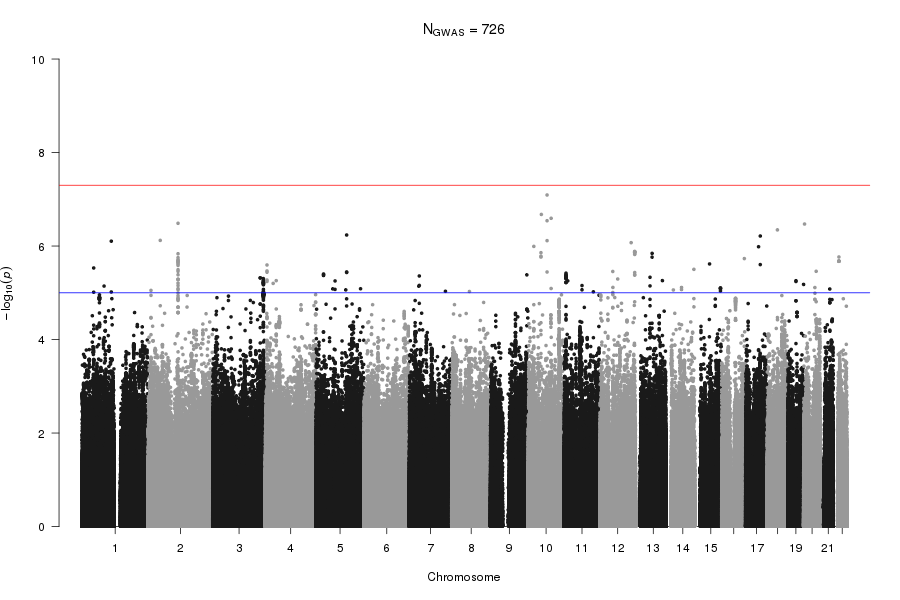

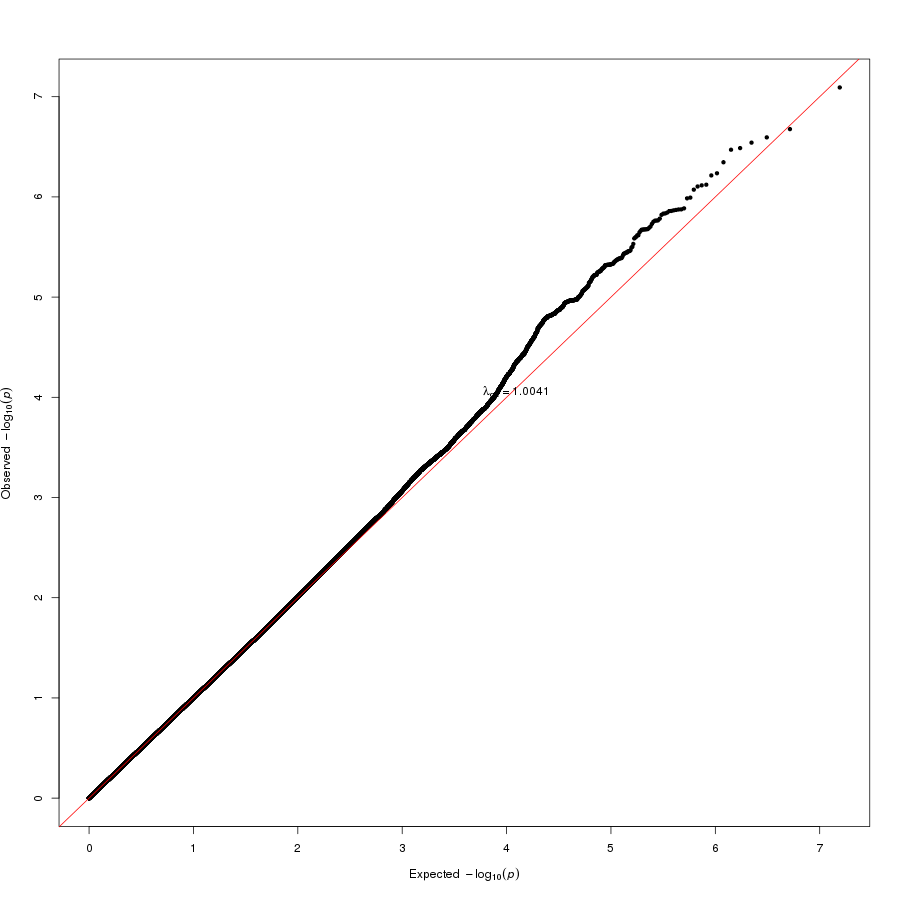


**Supplementary Figure 13B.** Ptau_ASSAY_Zscore


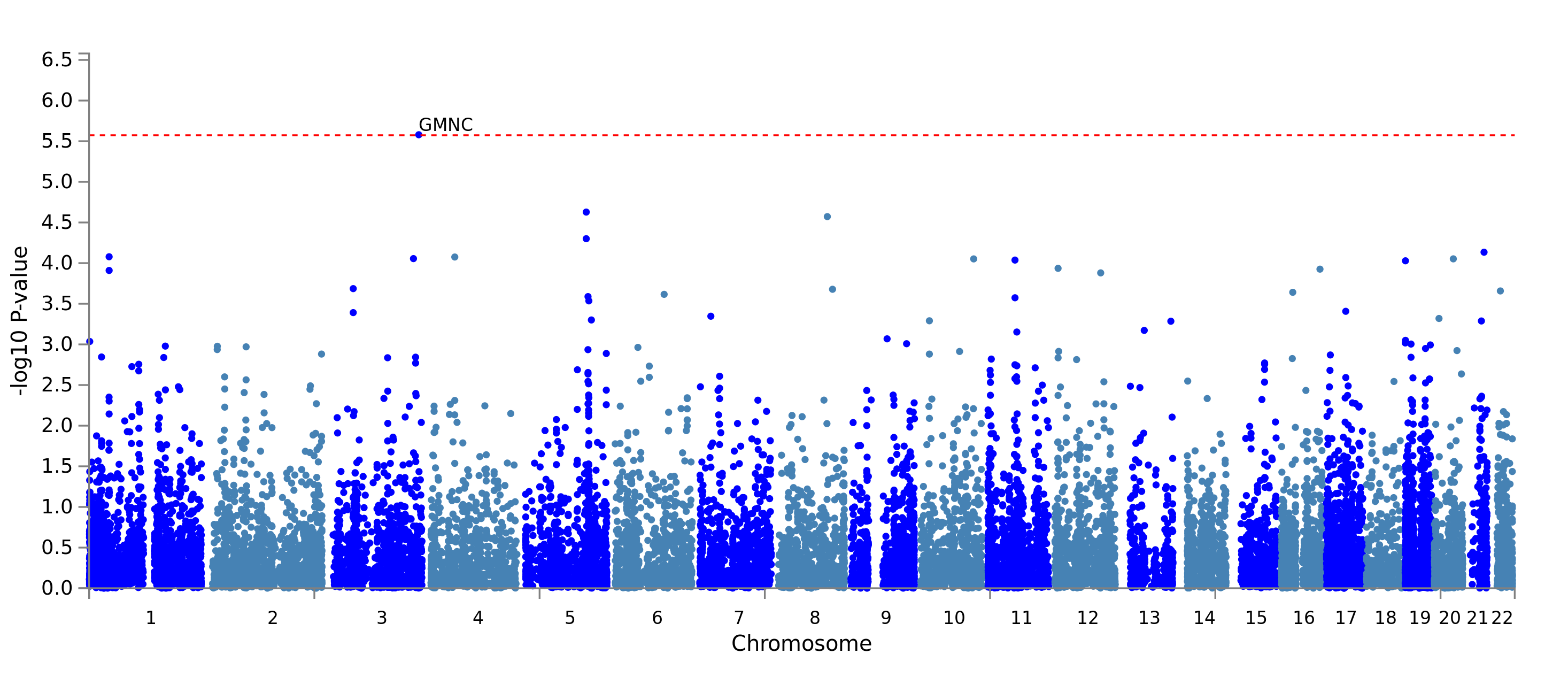

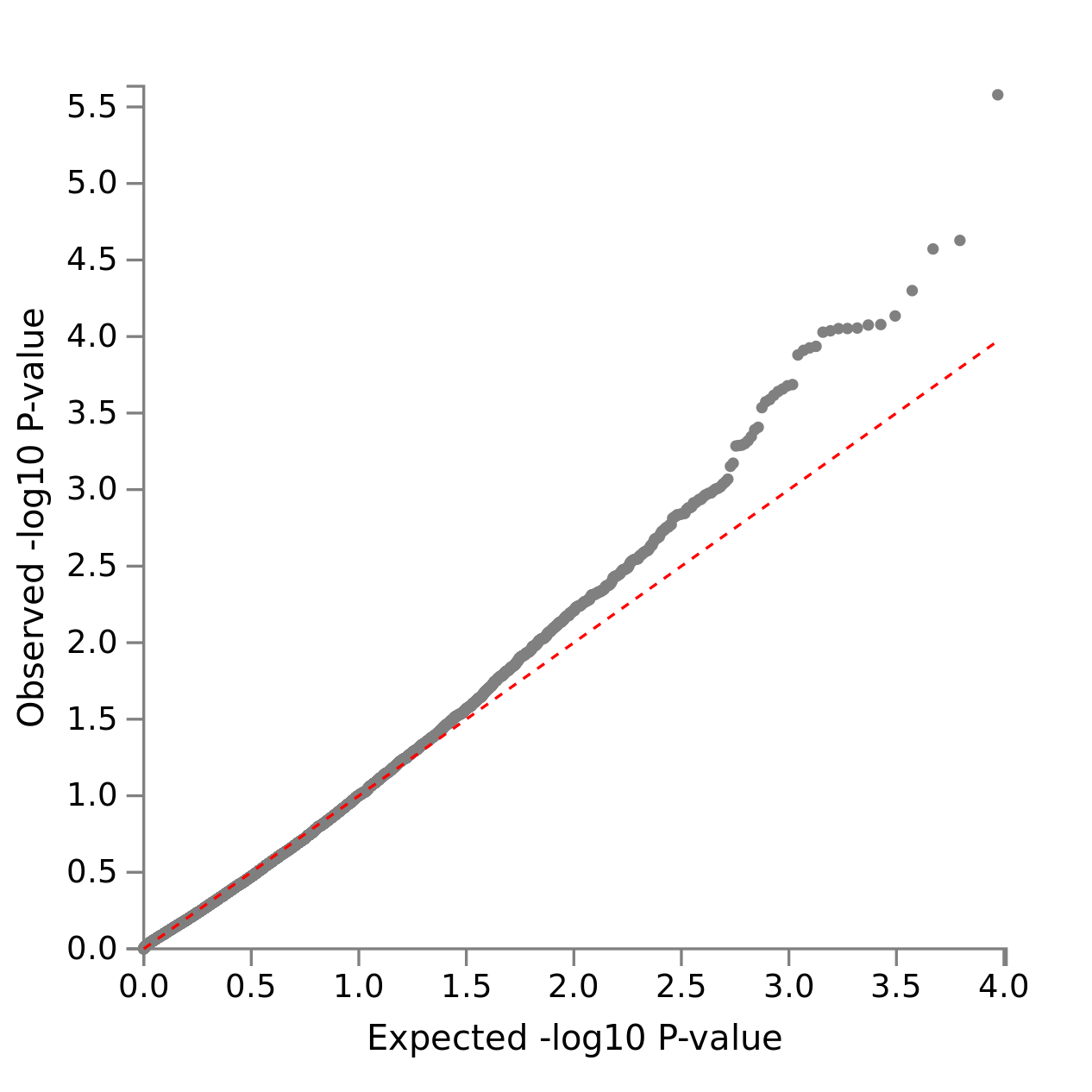

Supplement: Supplementary file 1 — Supplementary Materials [file 41398_2020_1074_MOESM1_ESM.doc]
